# Meanings of hope constructed by users of mental health services during the COVID-19 pandemic

**DOI:** 10.1590/1980-220X-REEUSP-2024-0182en

**Published:** 2025-03-28

**Authors:** Sarah Salvador Pereira, Angélica Martins de Souza Gonçalves, Simone Teresinha Protti-Zanatta, Maria do Perpétuo Socorro de Sousa Nóbrega, Bianca Cristina Ciccone Giacon, Sonia Regina Zerbetto

**Affiliations:** 1 Universidade Federal de São Carlos, Departamento de Enfermagem, Programa de Pós-graduação em Enfermagem, São Carlos, SP, Brazil.; 2 Universidade de São Paulo, Escola de Enfermagem, Programa de Pós-graduação em Enfermagem, São Paulo, SP, Brazil.; 3 Universidade Federal de Mato Grosso do Sul, Instituto Integrado em Saúde, Programa de Pós-graduação em Enfermagem, Campo Grande, MS, Brazil.

**Keywords:** Mental Health, Hope, COVID-19

## Abstract

**Objective:**

To analyze the production of meanings of hope in the discourses of specialized mental health services users in an inland city of Minas Gerais in the context of the COVID-19 pandemic.

**Method:**

Qualitative study based on the theoretical-methodological framework of French Discourse Analysis and the theoretical construct of hope. Semi-structured interviews were conducted from August 2022 to May 2023.

**Results:**

The discursive block entitled “The meanings of Hope constructed in the COVID-19 Pandemic” addresses the meanings constructed about hope that are permeated by the effects arising from discursive memories. The discursive excerpts enunciate signs of religious/spirituality discourse; solidarity discourse; disease model; and resilience discourse.

**Conclusion:**

Pre-constructed discourses elsewhere on spirituality/religiosity, solidarity, disease model and resilience permeate the production of meanings of hope in the discourses of mental health services users. It is important to enable nurses to grasp the constructed meanings of hope and to recognize them as a therapeutic resource that constitutes care.

## INTRODUCTION

The COVID-19 pandemic (Coronavirus Disease 2019), as an international public health emergency, was declared in 2020 by the World Health Organization (WHO) and triggered not only challenges to health systems and public health policies worldwide^(1)^, but also changes in all sectors of society, affecting working/employment conditions, education, and especially the population’s mental health.

Symptoms such as insomnia, stress, anxiety and depressive symptoms have been reported in the literature in the population in pandemic situations^(2)^. During the COVID-19 pandemic, the population experienced several difficulties that affected their mental health, such as: fear of this disease and death; fear related to a damaged source of income; sleep disorders; feelings of hopelessness, loneliness and depressive symptoms; anger, frustration or irritability; fear of the possibility of the individual or members of their family contracting COVID-19, anxiety, or other stress reactions^(3)^.

The entire society has suffered and continues to suffer from the consequences of the COVID-19 pandemic; however, some segments of the population were more exposed to the risks of COVID-19 and found themselves more vulnerable, such as people living with mental disorders or disorders related to the use of psychoactive substances (PAS)^(3,4)^.

In relation to people who consume PAS, the pandemic period influenced and intensified their clinical and psychosocial conditions, including an increase in their consumption^(5)^. Furthermore, the adherence and compliance of these users to treatment in health services was compromised due to social and economic changes, for example^(6,7,8)^.

It is assumed that, during the COVID-19 pandemic, the population needed hope to face daily challenges. It is believed that hope was even more compromised in the perception of users of specialized mental health services, due to factors of social isolation, changes in daily routine, difficulty in accessing care provided by mental health services, changes in the scope of work, doubts and uncertainties regarding the COVID-19 disease^(3)^.

Faced with this scenario of adversity and challenges, having and maintaining hope can be an important resource for promoting mental health and motivating to (re)build and lead life. The meaning of hope adopted in this study is that of vital, dynamic, complex and multidimensional force, a process characterized by a confident expectation of achieving a future goal that is significant to the person^(9)^. It is a psychosocial resource that promotes conditions for dealing with crisis situations and provides beneficial health effects. It is a support tool to address issues related to health needs and to maintain quality of life^(10)^.

In view of the foregoing, the scientific and social relevance of the theme of hope in mental health during the COVID-19 pandemic period is clear, especially in the context of alcohol and other drugs, and the question is: what are the meanings of hope constructed by users of specialized mental health services in the context of the COVID-19 pandemic?

The objective of this study was to analyze the production of meanings of hope in the discourses of users of specialized mental health services in an inland city of Minas Gerais in the context of the COVID-19 pandemic.

## METHOD

### Type

Qualitative, exploratory study guided by the tool *Consolidated Criteria for Reporting Qualitative Research* (COREQ). The study was based on the theoretical and methodological framework of French Discourse Analysis (DA) by Michel Pêcheux and his affiliate Eni Orlandi, who introduced the framework in Brazil, and on the theoretical interpretative framework of the Model of Hope by Dufault and Martocchio.

Discourse Analysis deals with the effects of meanings produced between speakers in their social locations determined by the social structure^(11)^. For DA, meanings are socially constructed and determined by history, ideology, and the unconscious^(12)^. DA allows us to interpret discourse by identifying traces and marks of the ideological, historical, and social context, which are not organized by the speaker him/herself, but which are revealed in the linguistic materiality^(13)^.

The meanings produced by the subjects are imprinted in the Discursive Formations (DF), which constitute something that, in a given position and situation, allows what can and should be said. Therefore, a given DF produced by the subject is interpellated by another DF constructed before and externally to him/her. In this configuration, we find forgetfulness number 1, a term used in DA to designate the illusion that we originate from thought or speech^(13)^.

Dufault and Martocchio’s Model of Hope^(9)^ argues that hope can be defined as a dynamic vital force, which involves several dimensions characterized by confident expectation of achieving a future objective that is significant for the person. The six dimensions of hope consist of: affective, cognitive, behavioral, contextual, affiliative and temporal^(9)^.

### Local

The research involved four specialized mental health services: Psychosocial Care Center for Alcohol and Drugs (CAPS AD), Psychosocial Care Center II, Multidisciplinary Mental Health Center and Post-Covid Mental Health Care Center, all in an inland city in the state of Minas Gerais.

### Population and Selection Criteria

We worked with a convenience sample of 14 users who use such specialized mental health services. The inclusion criteria were: age greater than or equal to 18 years and undergoing treatment in a specialized mental health service, and the exclusion criteria involved: presentation of difficulty in understanding that prevented the progress of the interviews and/or being intoxicated or having a psychotic episode on the day of the interview, presenting altered physical and psychological signs. In intoxication, depending on the PAS, there would be: intense mood lability, euphoria, drowsiness, motor incoordination, and mental confusion. In a psychotic outbreak, there would be: agitation, aggressiveness, mental confusion, hallucinations, or delusions.

The sample closure was due to theoretical data saturation, in which the saturation point is considered when there is an understanding that sufficient data has been obtained to understand the theme^(14)^.

### Data collection

Data collection was carried out from August 2022 to May 2023, by the study’s main researcher (doctoral student), who has experience in mental health, the theme of hope, and in interviews for qualitative research. A sociodemographic and clinical questionnaire was used to characterize the study participants (with questions about sex, religion, education, marital status, current employment situation, length of treatment in the service and satisfaction with the treatment) and a semi-structured interview script with inquiries that questioned the meaning of hope for users during the COVID-19 pandemic, future perspectives in their lives, and factors that inhibit and facilitate hope. The interviews lasted an average of 25 minutes and were audio recorded.

Participants were recruited through prior contact and referral by health professionals from the respective services, after authorization from users, or by the researcher in the waiting room. The interviews took place in these services, in a private room, ensuring privacy and following the health protocols and safety measures against COVID-19 determined at the time by the Ministry of Health.

Participants were identified as Discursive Subjects (*SD*) followed by sequential numbering according to the order of the interviews. The discursive sequences were represented by the acronym “sd”, followed by the numbering, with texts with the presence of linguistic traces and marks being underlined.

### Data analysis and treatment

The analytical devices of the DA methodological framework were used^(13)^, which allowed capturing what was said and what was not said beyond the surface and to analyze the materiality that conveys the discourse. The first stage consisted of transcribing the interviews, identifying traces and linguistic marks to understand the discursive functioning. The second stage allowed us to understand how meanings are constituted in a given discourse, identifying the discursive segments and cutouts. In the next stage, the discursive excerpts were grouped into discursive blocks, according to similar ideological formations. To validate and ensure the reliability of the discursive sequences, excerpts and discursive blocks, three nurse researchers participated: one on the theme of hope and mental health, another on the theme of alcohol and other drugs, and a third on DA.

### Ethical aspects

Study approved by the Human Research Ethics Committee of a Federal University, in 2022 under opinion no. 5.264316. The study complies with National Health Council Resolution No. 510/16. All participants signed the Free and Informed Consent Form.

## RESULTS

A total of 18 users were invited, of which 4 declined the invitation and there were no withdrawals once the interview began. Fourteen interviews were conducted with users of specialized mental health services, including 5 users from the Multidisciplinary Mental Health Center, 4 users from CAPS II, 3 users from CAPS AD, and 2 users from the Post-COVID Mental Health Care Center. Of these 14 individuals, 10 were women and 4 were men. The average age is 40 years. Regarding marital status, 7 were single, 2 were married, 4 were divorced, and 1 was a widow; 2 had higher education, 5 had finished high school, 4 had unfinished high school, 1 had finished primary education, and 2 had unfinished primary education. Of the users, 7 people were Catholic, 4 were Evangelical, 1 had no religion, 1 was a spiritualist, and 1 was agnostic. Currently, 11 individuals were not working and 3 were. The average time spent in the service was 18 and a half months.

Data analysis produced the discursive block entitled “The meanings of Hope constructed in the COVID-19 Pandemic”. This discursive block addresses the meanings constructed on hope that are permeated by the effects arising from discursive memories. In DA, interdiscourse is resumed through discursive memory; it is what has already been said previously, in another pre-constructed place, that makes the saying possible. The collective and social memories are the ones outlined in history and materialized in discourse^(13)^. The discursive excerpts reveal signs of religious/spiritual discourse, solidarity discourse, disease model, and resilience discourse.

Excerpts 1 to 4 highlight the discursive memories arising from religious/spirituality discourse, in which religious entities provide light, which reinforces the existence of God. There is the prospect of a better and transformative future, supported by faith, religion, and spirituality.

Excerpt 1: *[Hope] is to no longer do the things I used to do (sd8). It is not living in that dark world, see that everything is possible, that God exists (sd9). And that everything can work out (sd10).* (SD5).

Excerpt 2: *Jesus is everything (sd6). Jesus is hope (sd7). Did I answer you well? Hope is when we do the right thing and want to have faith (sd8).* (SD11).

Excerpt 3: *pray, intercede, tell people a little about God’s love, (sd10) God is love in our lives, (sd11) express a little bit of the spiritual part so that people do not lose hope either (sd12) and show them that nothing is lost, right? (sd13) it’s never too late to start over...you know, a new life (sd14).* (SD14).

Excerpt 4: *My hope comes from God, comes from Jesus (sd10). I can put faith with hope, almost the same word (sd11). I have faith that tomorrow will be better, and I have hope that tomorrow will be better, that everything will change (sd12).* (SD6).

Excerpts 5 to 7 highlight discourses supported by the Interdiscourse of solidarity, in which there is the expression of the idea that the COVID-19 pandemic inspired a feeling of solidarity in society, as well as affection, humbleness, and the feeling of valuing the things around.

Excerpt 5: *[Hope is] a green symbol, you know? (sd3) like, of solidarity... of more affection, more care... (sd4) people... they should be more like that (sd5). Nowadays... nowadays there are a lot of fights, a lot of disagreements... (sd6)*. (SD1).

Excerpt 6: *I feel like... that [the pandemic] is now improving, in certain aspects, things are getting back to normal (sd2) People are becoming humbler with each other. they are getting to know...(sd3) to know how to have God more in the heart...(sd4) give value to the things you lose...(sd5).* (SD4).

Excerpt 7: *Ah we imagined that people would change a little, that people would stop and step on the brakes a little (sd4), and to value life a little more, how people romanticized so much during the pandemic (sd5), but now that everything is back to normal everyone forgot, right (sd6), this romanticized view and unfortunately humanity is walking normally again (sd7).* (SD12).

Excerpts 8 to 11 state the effects of meaning affected by discursive memories arising from the disease model. The discourses were affected by the biomedical paradigm and the problem-solution model, pre-constructed ideas that were consolidated in a hegemonic manner in the history of health and care.

Excerpt 8: *On the pandemic, my condition worsened a lot (sd11). In the first place, I wait for my cure (sd12). And I know that in the name of Jesus, I will be healed (sd13). I will get myself free from all these medications, because they are no joke (sd14).* (SD7).

Excerpt 9: *I hope not to have any relapses (sd6). We can have relapses, you know, but I I’m reacting against (sd7).* (SD5).

Excerpt 10: *[In the pandemic] I was trafficking, so I had drugs in the house anyway, that’s when I used even more (sd1). I had nothing to do, so I stewed using even more (sd2). [Today I have a] Feeling that I have to live on medicine you know...because otherwise, it can’t hold up (sd3).* (SD9).

Excerpt 11: *Then I discovered that I was depressed, now depression is installed (sd6), she [the unit’s psychologist] already told me that it’s installed, so I have to do everything to get out (sd7), so it’s like a drug, you go, one day you’re fine, another day you’re not fine, right? (sd8) [...] I need to get out of this depression that I am in, to tell you next time that I have dreams, because I don’t have them anymore (sd10). Hope meant a lot to me, you know, hope for me was everything, it was a key word (sd12)[...]now honey, I’m going to tell you, in the depression that I am, as it is installed, if I tell you that I have a goal in life, I’ll be lying. (sd14).* (SD13).

Excerpts 12 to 15 express feelings of hope arising from discursive memories anchored in the discourse of resilience. The excerpts express feelings of valuing internal and personal resources to overcome adversity. Some speeches reiterate the idea that difficulties are important to bring inner strength.

Excerpt 12: *It [the pandemic] is being so, basically, an apprenticeship, you know? (sd7) it was like that, in the sense of... Sense of looking at things, you know? (sd8) that the pandemic for me, it wasn’t easy (sd9). It was a tremendous mess (sd10). So, it went well... yeah... there was an imbalance in my mental health (sd11) [...] but then I ended up getting up and... I changing the situation a little (sd12). [My hope] comes more even from my courage, so to speak. Sometimes I really have courage to live life more. Besides being quite discouraging, as sometimes happens, it’s... sometimes, I’m quite brave in doing some things (sd13).* (SD1).

Excerpt 13: *it wasn’t easy, it wasn’t easy for anyone, everyone suffered, the consequences of the pandemic (sd3), so we are survivors of a shipwreck...(sd4) we are still surviving, thank God...(sd5).* (SD14).

Excerpt 14: *So, I hope for happiness, run after all my goals (sd12). Wake up early and fight, have a normal life, like anyone else has (sd13).* (SD5).

Excerpt 15: *[Hope means] Trying to achieve the positive points in life... (sd9) “ah! I hope there won’t be a problem”... (sd10) but You have to have problems in life to grow...(sd11) I take [hope] from myself and a little bit from the faith I have, in God... (sd12) the fact is that my life was kind of complicated since I was a child, so I think it was me, then I needed to be stronger so I could fight (sd13).* (SD6).

## DISCUSSION

The study showed that the subjects produced different meanings about hope during the pandemic period, meanings that were affected by discursive memories, which are not consciously accessible to the individual, but are constitutive of the discourses^(12,13)^. The meanings are already in the world before, in the already-said, in the pre-constructed, and return in the saying^(15)^.

Excerpts 1 to 4 express feelings of hope permeated by discursive memories constructed by religious/spirituality discourse. Religiosity can be defined as the actions, practices, and beliefs linked to a particular religion. Spirituality has a broader meaning, involving the personal search for questions that are essential to life and the connection with the sacred or transcendental^(16)^. Excerpt 1 presents SD5’s response to the question - “How would you describe hope in your life?”. The conditions for producing the speeches are those of a user of the CAPS AD service, who has recently returned to treatment and is suffering the consequences of the abusive use of PAS. Excerpt 1 states that the sense of hope is the absence of drugs. In sd8 the expression “the things I did” silences the meaning “using drugs”, and from SD5’s perspective, such consumption is considered inconvenient or inappropriate. This silence consists of the concealment of other possible meanings that cause discomfort, that is, conceived as inconvenient^(13)^.

In a metaphorical effect of sliding senses, “dark world” means “drugs” and drugs blind the person and everything is impossible, while in the opposite sense, “light” would be the figure of God, that is, when the person comes out of the darkness, “sees” and “views” the existence of God. In this sense, drugs are darkness and God is light. The paradox between darkness and light refers to the religious discourse that the figure of God would be the solution for the PAS use problem. As SD5 states, when she sees God, “everything is possible” (sd9) and “everything can work out” (sd10).

In excerpt 2, the sense of hope is personified in the figure of Jesus. In a shift of meanings, the signifier “hope” takes on a spiritual and religious meaning. The discursive follow-up - “do the right thing” - silences other possible meanings that the right thing would be belief in Jesus and the will, the desire to have faith. This excerpt expressed signs of discursive memories articulated with religious and justice/ethics discourse, in which hope belongs to the universe of faith, of the transcendent, but of justice/ethics, that is, of doing the right thing.

Excerpt 3 presents SD5’s response to the question - “How would you describe hope in your life?” The sense of hope takes on the meaning that God helps people not to lose hope. The idea of hoping for a new life, a new beginning (future perspective) – emerges in the paraphrase “It’s never too late to start over” (sd14). sd11 reinforces the discourse of spirituality through the paraphrase “God is love in our lives”. The pre-constructed elsewhere returns in the saying, demonstrating meanings that spirituality expresses senses of love, charity, help, and hope.

SD14 states that hope in her life would be to share this meaning with other people. The subject lists actions of a spiritual nature that for her are related to the sense of hope (praying, interceding, talking about God to people). These actions are part of the behavioral dimension of hope in the Model of Hope, which involves the social, psychological, physical or religious actions involved in the process of waiting and having hope^(9)^.

Excerpt 4 presents SD5’s response to the question: “How would you describe hope in your life?” In metaphorical effect, the sense of hope is shifted to the sense of faith, in a movement of contextual transfer. Faith and hope, for SD6, are “almost” synonymous, that is, for her the sense of hope is closely connected with the belief in spirituality. Paraphrasing Dufault and Martocchio’s Model of Hope^(9)^, there is a sense of generalized hope, that is, not referring to a specific object of hope, but rather a generalized feeling of a “better tomorrow”, of a “better future” and the expectation of changes in the present.

Religious/spirituality discursive productions exemplify how the affiliative dimension of the hope process is mobilized^(9)^, which refers to relationships with other people (living or dead) or with a Higher Being. The discursive excerpts state that relating to God constitutes a source of hope for the subjects of the discourse. Spirituality is part of the behavioral dimension of hope^(9)^, considering that religious rites and practices, such as prayers, meditations, worship and participation in religious institutions, for example, are actions and behaviors that can facilitate the emergence or strengthening of the state of hope.

Studies^(17,18)^ corroborate the assumption that the dimension of spirituality is closely related to the experience of hope and demonstrate that spiritual beliefs are significant internal resources for obtaining or maintaining hope. Spirituality was identified as a source of hope in these studies^(17,18)^.

The speeches above evoke discursive memories arising from religious/spirituality discourse. The meanings built on hope are strongly permeated by the meanings of faith, belief, and spirituality. As a condition for broad production, it is worth noting that the Brazilian population is mostly Christian, of Catholic or Evangelical religions^(19)^. The conditions of production refer to the circumstances in which discourses are produced and their relationship to enabling speech. They involve the socio-historical context, imaginary, and ideological aspects in which the subject is inserted^(13)^.

Religion presents itself as a specific form of language that has its own codes and rites and acts as a mediator in the process of giving meaning to life and reality^(16)^. Religions, as social practices, are part of the constitution of the subject and social organization, influencing the subjects’ way of being and constructing meanings^(16,20)^, therefore it is expected that spirituality will be transversal to the discourses of the discursive individuals of this study.

Another discourse highlighted in this study involved the call for solidarity as a way of dealing with the difficulties imposed by the pandemic. The dissemination conveyed by social networks and media discourses was that the pandemic provided a positive sensation, that is, the pandemic could invoke the experience and feelings of solidarity in people, as well as the perception that the pandemic adversity would trigger feelings of giving more value to people’s lives.

The discourse of solidarity is resumed through paraphrasing processes in discursive excerpts 5 to 8. Excerpt 5 is part of the answer given by SD1 to the question: “When you hear the word hope during the pandemic, what comes to mind?” In a metaphorical effect, SD1 rescues the discursive memory that the green color symbolizes hope. The green color, in the social imagination, is related to nature, germination, and rebirth; preserving the environment, balance, and harmony (contrary to an environment of fighting and discord). The discursive segments “it’s like, of solidarity... of more affection, more kindness… (sd4) people... they should be more like this (sd5). Nowadays... nowadays there are a lot of fights, a lot of disagreements... (sd6)” reveal signs of a sense of hope related to the sense of solidarity. There is a relationship of meaning effects anchored in the Interdiscourse of valuing solidarity, that is, being supportive involves being affectionate and caring, as well as maintaining a harmonious environment. The subject attributes the meaning of solidarity to the signifier “hope”.

Excerpt 6 also enunciates meanings anchored in the Interdiscourse of solidarity, in which the pandemic makes people more supportive and more appreciative of the things they lose. For SD4, hope during the pandemic period would be to have more “humbleness” among people. A Freudian slip is perceived when SD4 chooses the signifier “humbleness” and not other possible ones, such as “humanity or solidarity”. However, in the discursive excerpt, the signifier “humbleness” acquires another meaning when inscribed in this specific discursive formation - the sense of solidarity, a meaning different from its literal meaning which, according to the Aurélio dictionary, means “modesty, simplicity”. For DA, this effect of meaning demonstrates how words do not have a literal, original meaning; the meaning can be displaced and receive another effect, that is, circulate between different discursive regions^(21)^.

Solidarity emerges as society’s response to the pandemic period, which constitutes a resource that can be mobilized individually and/or collectively, by companies, groups organized by civil society, social movements and public authorities^(22)^. In the study, the meaning of “solidarity” is related to the meaning of “philanthropic solidarity”, expressed by individual or small group conduct to carry out voluntary, welfare and charitable actions, as well as maintaining an empathetic stance.

The signifier “hope”, when belonging to the semantic universe of the signifier “solidarity”, refers to the affiliative dimension of the Model of Hope^(9)^. Mutual help, reciprocity, and concern for others are part of the process of hope as we relate to each other and we can strengthen hope through relationships.

Excerpt 7 expresses the sense that the pandemic would trigger good for humanity, that is, reflective moments for people to stop and take life “slower”. According to SD12, there was a romanticization during the pandemic period, that is, experiencing difficult times can encourage people to value life more. In the effect of a sliding of meanings, in sd4 “people should stop and step on the brakes a little” gives effects of deceleration, calm, pause, which, in the given situation, would imply the appreciation of life (sd5). However, in sd6, SD12 concludes that this expected change did not occur, when “everything returned to normal”, “humanity moves forward as normal”, that is, it returns to an accelerated pace, a pace that does not provide the appreciation of life. Another important clue in this discursive excerpt is the “romanticized vision” mentioned in sd7, which refers to the discursive memories disseminated during the pandemic, of a good period for humanity, that is, of valuing life in the face of pandemic adversities.

During the pandemic period, online social networks and media exposed the diverse social imaginary, that is, part of the population favored disseminating information about the pandemic, another part emphasized the dangerous potential of the virus and the losses caused by complications of COVID-19, and another expressed positive experiences arising from the pandemic, such as reports of solidarity and charitable acts, individual experiences of overcoming and positive personal experiences of reinventing habits and routines, according to literature^(23)^.

Excerpts 8 to 11 state the effects of meaning affected by discursive memories arising from the disease model, in which symptoms and medications acquire central positions in the therapeutic process, based on the premise of “disease-cure” and “problem-solution”. In the context of the use of alcohol and other drugs, chemical dependency is a disease and its “cure” is only through abstinence^(24)^. In the context of mental disorders and those derived from the consumption of PAS, mental disorder is a disease that can be treated through medication, constituting a problem that has a cause and requires a solution, in this case, the medicine.

The rationalist “problem-solution” paradigm was hegemonic until the first experiences of the Psychiatric Reform in the 1960s and supported practices in Mental Health and Psychiatry. In this paradigm, the given problem would be the disease and the expected solution would be the cure. Therapy in this logic is considered a process that links the diagnosis to the expected prognosis – the cure. With the first Reform initiatives and the advancement of knowledge in the area of mental health, it is understood that people with mental disorders have an integral, complex, and concrete experience that cannot be reduced to the “problem-solution” logic.^(25)^. To work in practice with users of specialized mental health services, it is necessary to overcome the problem-solution paradigm and move towards a meaning that, in the context of mental disorders, psychological suffering is a subjective and complex experience in a social context and, in the context of alcohol and drug consumption, there are approaches that recognize the autonomy, protagonism, and uniqueness of users, such as the Harm Reduction approach^(25,26)^. This way, users are considered social subjects and actors of change and reconstitution of meanings in life^(25)^.

In excerpt 9, SD5 is hopeful of not having any more “relapses”, and this signifier reveals signs of a discourse based on the disease model and relapse prevention. From this perspective, PAS dependence is a disease and, therefore, the aim is to achieve a cure, that is, abstinence. Relapse means going back to use, which must be prevented in this model^(24)^.

The excerpts above express the cognitive dimension of the process of waiting and having hope, when the discursive subjects elaborate, interpret, judge, and list their objects of hope, defining priorities, for example^(9)^. SD2 attributes the meaning of hope to the desire for “recovery” and SD7 highlights healing as its main and first object of hope. SD5 defines abstinence from PAS as his object of hope, remaining free from relapses.

In excerpt 10, sd1 points out contradiction and criticism regarding the position of the discursive subject in his social position, at a certain moment in his life, that is, being a drug dealer and a consumer of PAS, at the same time. The signifier “stewed” in sd2 denotes senses of ebullience, effervescence, bubbling, which enunciates meanings of intense drug use, especially during the pandemic. It stands out in sd3 - “I have to live on medication” - the statement of meanings takes up the discursive memories of the disease model, in which medications are the main therapeutic solution for psychological suffering^(24)^. The speech highlights not only the dependence on PAS, but also on drugs, which supports the speaker’s management of his life.

In excerpt 11, SD13 says she has no hope due to the “setting” depression, with this signifier being a metaphorical effect of contextual transfer, that is, meanings of “realized”, “settled”, “diagnosed”. For SD13, the fact that her mental disorder is “installed” conditions and determines her experience of hope. The linguistic marks and traces derive metaphorical effects from the fact that the user would have to “do everything to get out” of depression, to cure herself, so that in the future, “next time”, she could have dreams and goals.

The condition of the diagnosis of depression, or the lack of diagnosis, is decisive for the ability to have hope. The contextual dimension influences the process of hope, as life circumstances and situations interfere with the subject’s ability to maintain hope^(9)^. The condition of strict production of worsening depression and the experience of mourning by SD13 due to the pandemic triggered feelings of hopelessness.

The assumption that hopelessness and hope are not antagonistic reinforces the perception that the same situation can provoke opportunities for hope or hopelessness, depending on how the individual perceives, interprets, and reacts to the context. Contexts denoting hopelessness can awaken new goals, review plans and its strategies^(9)^.

Excerpts 12 to 15 express feelings of hope that are affected by pre-constructed discursive memories of the resilience discourse. SD1 reports “mental health imbalance” when defining the personal meaning of hope in pandemic times. The signifier “imbalance” denotes the metaphorical effects of the meanings that psychic suffering makes her lose her balance and fall, although the discursive subject recognizes his/her “courage” as a source of motivating hope to “get up” and “change the situation”. These meanings point to the appreciation of internal characteristics and personal resources for coping and resilience, seeking to overcome the difficulties of the pandemic period.

Recalling the strict production conditions of SD1 in which the speech was enunciated, the interviewee’s non-verbal language stands out, expressed by her tired and discouraged posture. There is contradiction and ambivalence in the face of the recognition of SD1’s “courage” and the subject position of discouragement. Hope is closely linked to the condition of uncertainty and is characterized as a human experience that can oscillate, alternating between moments of high and low hope. Some affective responses to uncertainty are people’s feelings of anxiety, vulnerability, worry, and sadness^(9)^.

In discursive excerpt 13, SD14 uses the metaphorical figure of speech to compare society to a sinking ship during the pandemic, denoting feelings of destruction and annihilation, but with survivors. The discursive subject emphasizes empathy, when enunciating the suffering shared and collectively experienced by people during this period. The selection of the signifier “survivors” in turn suggests meanings of resilience. In the strict production condition in which the speech was delivered, SD14 believes that it is possible to adapt and overcome the difficulties imposed by the pandemic, that is, to “survive the shipwreck”.

In excerpt 14, the subject of the discourse attributes to the signifier “hope” meanings of resilience and effort, elucidating the functioning of the behavioral dimension of hope, which involves the actions mobilized by the subjects to achieve something significant for themselves and for others^(9)^. The discursive subject develops strategies related to the field of action, of acting (waking up early, running after things, fighting), which will help him achieve his expected result. sd13 states the linguistic mark of paraphrase, when SD5, in his saying - “Wake up early and fight” - paraphrases the popular saying - “The early bird catches the worm”. The paraphrase mechanism resumes pre-constructed meanings of valuing dedication and effort.

For SD5, such meanings are attributed to “having a normal life”, when choosing the signifier “normal” and not others. This DA phenomenon is called forgetfulness no. 2, in which the subject of the discourse privileges and selects certain sayings to the detriment of others^(12)^. The selection of a certain word to the detriment of another one has a meaning that is related to the broad and strict conditions of production, to the historical and ideological^(13)^. “Normal” for SD5 is about hard work, functionality, and struggle.

It is clear that the meaning of resilience is transversal to the above excerpts, which is understood as a complex phenomenon, which has been studied over the last few years by several areas, such as Human Sciences and Health Sciences and conceptualized by several authors^(27,28)^. Resilience can be defined as the process of facing challenges or adversities and as the ability to develop adaptive responses to obtain expected results. It is a process developed throughout life that involves personal, interpersonal, and social coping resources^(29)^. The sense of resilience, pre-constructed previously and elsewhere, is taken up by the subjects and materialized in their speeches, when they describe their experience of hope during the COVID-19 pandemic.

In excerpt 15, the meaning revealed is to value overcoming adversity, when answering the question: “What does hope mean to you?” The meaning highlighted is that of resilience and overcoming, as the subject mobilizes internal resources (personal strength/resilience) and external resources (a little bit of faith) to empower themselves in the face of adversity. sd11 - “you have to have problems in life to grow” - presents the linguistic clue of paraphrase, understood as a return of memory in the saying^(13)^. This discursive sequence takes up meanings pre-constructed elsewhere that - “suffering makes people grow” and that difficulties enable learning and positive changes.

For DA, when investigating the constitution of a discourse, the discourses that are inserted in it, that is, the Interdiscourse^(30)^, are analyzed. sd11 takes up the socially disseminated discourse, therefore, of ideological formations of valuing adversity. For the subject, hope acquires the meaning of overcoming life’s difficulties and sd13 - “I needed to be stronger to be able to fight” - reinforces the value of individual effort, ability and competence to face adversity.

For DA, production conditions refer to a sum of variables that make up the situation in which the speech is being emitted, considering the socio-historical context, personal context and the place from which the subject speaks^(30)^. In this excerpt, it can be seen that in the face of a macro context (pandemic) and micro context (personal life story), the subject experienced difficult life situations - “my life has been a bit complicated since I was a child” - driving him to “fight” against adversities.

It is interpreted that the ability to learn from facing the problem in the past or present supports a future resolution of the problem, but not its exclusion in the past, present, and future. Therefore, the goal is to hope for competence and ability to solve future problems. Thus, such a context involves the temporal dimension of hope, in which the experience of the past and present subsidizes the future, to enable competence and ability^(9)^.

Regarding the limitations of this study, they may be related to the period of data collection concerning the time of the COVID-19 pandemic wave, considering the strict production conditions in which vaccination was initiated and the end of this disease, as a public health emergency. Another limitation corresponds to the predominance of female participants in the study in relation to the perception of the meaning of hope, requiring expansion of the study to include the male population.

The results of this study contribute to a more empathetic and sensitive listening to the experience of hope arising from the discourses of users of specialized mental health services during the COVID-19 pandemic period, which can help both health professionals, especially nurses, and users of mental health services, to (re)cognize hope in themselves and in others as sources that promote mental health.

## CONCLUSION

The discourses produced about hope evoke discursive memories constructed previously and externally to the subject. The meanings of hope were permeated by the religious/spirituality, solidarity, of disease model, and resilience speeches. It is believed that it is important to train health professionals, especially nurses, who work in CAPS and CAPS AD, to understand the constructed meanings of hope and to recognize them as a therapeutic resource that constitutes care. The importance of recognizing the promotion of hope as an intervention in clinical and care practice is highlighted.
