# Supplementary material for: Meanings of hope constructed by users of mental health services during the COVID-19 pandemic
Source: Rev Esc Enferm USP. 2025 Mar 28;59:e20240182. doi: 10.1590/1980-220X-REEUSP-2024-0182en (PMC11968133; doi:10.1590/1980-220X-REEUSP-2024-0182en)
Supplement: Translation

# Translation: Sentidos da Esperança construídos por usuários de serviços de saúde mental na pandemia da COVID-19

**DOI:** 10.1590/1980-220X-REEUSP-2024-0182pt

**Authors:** Sarah Salvador Pereira, Angélica Martins de Souza Gonçalves, Simone Teresinha Protti-Zanatta, Maria do Perpétuo Socorro de Sousa Nóbrega, Bianca Cristina Ciccone Giacon, Sonia Regina Zerbetto

**Affiliations:** 1 Universidade Federal de São Carlos, Departamento de Enfermagem, Programa de Pós-graduação em Enfermagem, São Carlos, SP, Brasil.; 2 Universidade de São Paulo, Escola de Enfermagem, Programa de Pós-graduação em Enfermagem, São Paulo, SP, Brasil.; 3 Universidade Federal de Mato Grosso do Sul, Instituto Integrado em Saúde, Programa de Pós-graduação em Enfermagem, Campo Grande, MS, Brasil.

**Keywords:** Saúde Mental, Esperança, COVID-19

## Abstract

**Objetivo:**

Analisar a produção de sentidos da esperança nos discursos de usuários de serviços especializados de saúde mental de uma cidade do interior mineiro no contexto pandêmico da COVID-19.

**Método:**

Estudo qualitativo embasado no referencial teórico-metodológico da Análise de Discurso de matriz francesa e constructo teórico de esperança. Foram conduzidas entrevistas semiestruturadas no período de agosto de 2022 a maio de 2023.

**Resultados:**

O bloco discursivo intitulado “Os sentidos da Esperança construídos na Pandemia da COVID-19” aborda os sentidos construídos sobre esperança que são permeados pelos efeitos advindos de memórias discursivas. Os recortes discursivos enunciam indícios do discurso religioso/de espiritualidade; discurso de solidariedade; modelo de doença; e discurso de resiliência.

**Conclusão:**

Os discursos pré-construídos, em outro lugar, sobre espiritualidade/religiosidade, solidariedade, modelo de doença e resiliência permeiam a produção de sentidos da esperança nos discursos de usuários de serviços de saúde mental. É importante capacitar os enfermeiros a apreenderem os sentidos construídos de esperança e a reconhecerem-nos enquanto recurso terapêutico constituinte do cuidado.

## INTRODUÇÃO

A pandemia da COVID-19 (Doença do Coronavírus de 2019), enquanto emergência de saúde pública internacional, foi decretada no ano de 2020 pela Organização Mundial da Saúde (OMS) e desencadeou não só desafios aos sistemas de saúde e às políticas de saúde pública mundialmente^(1)^, mas também modificações em todos os setores da sociedade, afetando as condições de trabalho/emprego, educação e principalmente a saúde mental da população.

Sintomas como insônia, estresse, ansiedade e sintomas depressivos foram relatados na literatura na população em situações pandêmicas^(2)^. Na pandemia da COVID-19, a população experenciou diversas dificuldades que afetaram a saúde mental, tais como: medo desta doença e da morte; medo relacionado à fonte de renda prejudicada; alterações do sono; sentimentos de desesperança, solidão e sintomas depressivos; raiva, frustração ou irritabilidade; medo da possibilidade de o indivíduo ou de membros de sua família contraírem a COVID-19, ansiedade ou outras reações de estresse^(3)^.

Toda a sociedade sofreu e ainda sofre com as consequências da pandemia da COVID-19; no entanto alguns segmentos da população estiveram mais expostos aos riscos da COVID-19 e encontraram-se mais vulneráveis, como pessoas convivendo com transtornos mentais ou transtornos relacionados ao uso de substâncias psicoativas (SPA)^(3,4)^. Em relação às pessoas que consomem SPA, o período pandêmico influenciou e intensificou as suas condições clínicas e psicossociais, inclusive no aumento deste consumo^(5)^. Além disso, a aderência e adesão desses usuários ao tratamento em serviços de saúde ficaram comprometidas devido a mudanças sociais e econômicas, por exemplo^(6,7,8)^.

Pressupõe-se que, durante a pandemia da COVID-19, a população necessitou de esperança para enfrentar os desafios diários. Acredita-se que a esperança esteve ainda mais comprometida na percepção de usuários de serviços especializados em saúde mental, devido a fatores de isolamento social, mudanças na rotina diária, dificuldade de acesso ao cuidado ofertado pelos serviços de saúde mental, modificações no âmbito do trabalho, dúvidas e incertezas quanto à doença da COVID-19^(3)^.

Diante desse cenário de adversidades e desafios, ter e manter a esperança pode ser um recurso importante de promoção da saúde mental e motivador para (re)construir e conduzir a vida. O sentido de esperança adotado neste estudo é o de força vital, dinâmica, complexa e multidimensional. Processo caracterizado por uma expectativa confiante de se alcançar um objetivo futuro significativo para a pessoa^(9)^. É um recurso psicossocial que promove condições para lidar com situações de crise e proporciona efeitos favoráveis à saúde. Constitui em ferramenta de suporte para enfrentar questões relacionadas às necessidades de saúde e manter a qualidade de vida^(10)^.

Diante do exposto, percebe-se a relevância científica e social da temática esperança na saúde mental durante o período pandêmico da COVID-19, principalmente no contexto de álcool e outras drogas e questiona-se: quais os sentidos da esperança construídos pelos usuários de serviços especializados de saúde mental no contexto da pandemia da COVID-19?

O objetivo deste estudo foi analisar a produção de sentidos da esperança nos discursos de usuários de serviços especializados de saúde mental de uma cidade do interior mineiro no contexto pandêmico da COVID-19.

## MÉTODO

### Tipo

Estudo qualitativo, exploratório e norteado pela ferramenta *Consolidated Criteria for Reporting Qualitative Research* (COREQ). O estudo foi fundamentado no referencial teórico e metodológico da Análise de Discurso (AD) de matriz francesa de Michel Pêcheux e de sua filiada Eni Orlandi, introdutora do referencial no Brasil, e no quadro teórico interpretativo do Modelo de Esperança de Dufault e Martocchio.

A Análise de Discurso ocupa-se dos efeitos de sentidos produzidos entre locutores em seus locais sociais determinados pela estrutura social^(11)^. Para a AD os sentidos são construídos socialmente e determinados pela história, pela ideologia e pelo inconsciente^(12)^. A AD permite interpretar o discurso identificando os vestígios e marcas do contexto ideológico, histórico e social, que não são organizados pelo próprio enunciador, mas que se revelam na materialidade linguística^(13)^.

Os sentidos produzidos pelos sujeitos imprimem-se nas Formações Discursivas (FD), as quais constituem algo que, em uma posição e conjuntura dadas, permite o que se pode e deve se dizer. Portanto, uma determinada FD produzida pelo sujeito é interpelada por outras FD construídas anterior e externamente a ele. Nessa configuração, encontra-se o esquecimento número 1, termo utilizado na AD para designar a ilusão de que somos originários do pensamento ou do dizer^(13)^.

O modelo de Esperança de Dufault e Martocchio^(9)^ defende que a esperança pode ser definida como força vital dinâmica, que envolve diversas dimensões caracterizadas por expectativa confiante de se alcançar um objetivo futuro significativo para a pessoa. As seis dimensões da esperança consistem em: afetiva, cognitiva, comportamental, contextual, afiliativa e temporal^(9)^.

### Local

A pesquisa envolveu quatro serviços especializados de saúde mental: Centro de Atenção Psicossocial Álcool e Drogas (CAPS AD), Centro de Atenção Psicossocial II, Centro Multiprofissional de Saúde Mental e Centro de Atendimento em Saúde Mental Pós-covid, pertencentes a uma cidade do interior de Minas Gerais.

### População e Critérios de Seleção

Trabalhou-se com amostra de conveniência de 14 usuários que utilizam tais serviços especializados de saúde mental. Os critérios de inclusão constituíram em: idade maior ou igual a 18 anos e fazer tratamento no serviço especializado de saúde mental e os critérios de exclusão envolveram a apresentação de dificuldade de compreensão que impediu o andamento das entrevistas e/ou estar intoxicado ou em surto psicótico no dia da entrevista, apresentando sinais físicos e psíquicos alterados. Na intoxicação, dependendo da SPA, seriam: labilidade intensa do humor, euforia, sonolência, descoordenação motora e confusão mental. No surto psicótico seriam: agitação, agressividade, confusão mental, alucinações ou delírios.

O fechamento amostral deu-se por saturação teórica de dados, em que se considera o ponto de saturação quando houver o entendimento de que já foram obtidos dados suficientes para compreender a temática^(14)^.

### Coleta de Dados

A coleta de dados foi realizada no período de agosto de 2022 a maio de 2023, pela pesquisadora principal do estudo (doutoranda), que possui experiência em saúde mental, na temática de esperança e em entrevistas para pesquisas qualitativas. Utilizou-se um questionário sociodemográfico e clínico para caracterização dos participantes do estudo (com perguntas sobre sexo, religião, escolaridade, estado civil, situação de trabalho atual, tempo de tratamento no serviço e satisfação em relação ao tratamento) e um roteiro semiestruturado de entrevista com perguntas que questionavam o sentido da esperança para os usuários durante o período da pandemia da COVID-19, perspectivas futuras na vida destes, fatores inibidores e facilitadores de esperança. As entrevistas tiveram duração média de 25 minutos e foram gravadas por áudio.

O recrutamento dos participantes foi por intermédio de contato prévio e indicação pelos profissionais de saúde dos respectivos serviços, após autorização dos usuários, ou pela pesquisadora em sala de espera. As entrevistas ocorreram nestes serviços, em sala privativa, assegurando a privacidade e respeitando os protocolos sanitários e medidas de segurança contra a COVID-19 determinados na época pelo Ministério da Saúde.

Os participantes foram identificados como Sujeitos Discursivos (SD) seguidos por numeração sequencial à ordem das entrevistas. As sequências discursivas foram representadas pela sigla “sd”, seguidas da numeração, sendo sublinhados os textos com presença de vestígios e marcas linguísticas.

### Análise e Tratamento dos Dados

Foram utilizados os dispositivos analíticos do referencial metodológico da AD^(13)^, os quais possibilitaram captar o dito e o não dito para além da superfície e analisar a materialidade que veicula o discurso. A primeira etapa consistiu em transcrever as entrevistas, identificar vestígios e marcas linguísticas para compreender o funcionamento discursivo. A segunda etapa permitiu compreender como se constituem os sentidos em determinado discurso, identificando os segmentos e recortes discursivos. Na próxima etapa, os recortes discursivos foram agrupados em blocos discursivos, de acordo com as formações ideológicas semelhantes. Para validação e fidedignidade das sequências discursivas, dos recortes e blocos discursivos, participaram três pesquisadores enfermeiros: um na temática de esperança e saúde mental, outro no tema de álcool e outras drogas e um terceiro em AD.

### Aspectos Éticos

Estudo aprovado pelo Comitê de Ética de Pesquisa com Seres Humanos de uma Universidade Federal, no ano de 2022 sob o parecer n. 5.264316. O estudo está em conformidade com a Resolução Conselho Nacional de Saúde n. 510/16. Os participantes assinaram o Termo de Consentimento Livre e Esclarecido.

## RESULTADOS

Foram convidados 18 usuários no total, destes 4 recusaram o convite e não houve desistência uma vez iniciada a entrevista. Foram realizadas 14 entrevistas com usuários de serviços especializados em saúde mental, sendo 5 usuários do Centro Multiprofissional de Saúde Mental, 4 usuários do CAPS II, 3 usuários do CAPS AD e 2 usuários do Centro de Atendimento em Saúde Mental Pós-COVID. Destes 14 sujeitos, 10 eram do sexo feminino e 4 do sexo masculino. A média de idade é de 40 anos. Em relação ao estado civil, 7 eram solteiros, 2 casados, 4 divorciados e 1 viúva; 2 possuíam ensino superior, 5 ensino médio completo, 4 ensino médio incompleto, 1 ensino fundamental completo e 2 ensino fundamental incompleto. Dos usuários, 7 pessoas eram católicas, 4 evangélicas, 1 sem religião, 1 espírita e 1 agnóstica. Atualmente 11 sujeitos não estavam exercendo atividade profissional e 3 sim. A média de tempo em acompanhamento no serviço foi de 18 meses e meio.

A análise de dados produziu o bloco discursivo intitulado “Os sentidos da Esperança construídos na Pandemia da COVID-19”. Este bloco discursivo aborda os sentidos construídos sobre esperança que são permeados pelos efeitos advindos de memórias discursivas. Na AD, o interdiscurso é retomado por meio de memória discursiva, é o já dito anteriormente, em outro lugar pré-construído, que possibilita o dizer. São as memórias coletivas e sociais que são delineadas na história e materializam-se no discurso^(13)^. Os recortes discursivos enunciam indícios de discurso religioso/de espiritualidade, discurso de solidariedade, modelo de doença e discurso de resiliência.

Os recortes 1 a 4 evidenciam as memórias discursivas advindas do discurso religioso/de espiritualidade, em que as entidades religiosas proporcionam luz, o que reforça a existência de Deus. Há perspectiva de um futuro melhor e transformador, apoiado pela fé, religião e espiritualidade.

Recorte 1: *[Esperança] é não fazer mais as coisas que eu fazia (sd8). Não viver naquele mundo escuro, enxergar que tudo é possível, que Deus existe (sd9). E que tudo pode dar certo (sd10).* (SD5).

Recorte 2: *Jesus é tudo (sd6). A esperança é Jesus (sd7). Respondi bem procê? A esperança é a gente fazer a coisa certa e querer ter fé (sd8).* (SD11).

Recorte 3: *orar, interceder, falar um pouco pras pessoas do amor de Deus, (sd10) Deus é amor nas nossas vida, (sd11) expressar um pouquinho da parte espiritual pra que as pessoas também não venham a perder a esperança (sd12) e mostrar pra eles que nada está perdido né, (sd13) nunca é tarde pra recomeçar...entendeu, uma nova vida (sd14).* (SD14).

Recorte 4: *A minha esperança vem de Deus, vem de Jesus (sd10). Eu posso colocar a fé com a esperança, quase a mesma palavra (sd11). Eu tenho fé que o amanhã vai ser melhor, e eu tenho esperança que o dia de amanhã vai ser melhor, que tudo vai mudar (sd12).* (SD6).

Os recortes de 5 a 7 evidenciam discursos amparados no Interdiscurso da solidariedade, em que há a expressão da ideia de que a pandemia da COVID-19 inspirou o sentimento de solidariedade na sociedade, bem como de afeto, humildade e o sentimento de valorizar as coisas ao redor.

Recorte 5: *[Esperança é] um símbolo verde, sabe? (sd3) assim, de solidariedade... de mais afeto, mais carinho... (sd4) as pessoas... elas deveriam ser mais assim (sd5). Hoje em dia... hoje em dia tem muita briga, muita discordância... (sd6).* (SD1).

Recorte 6: *Eu sinto que... que [a pandemia] agora tá melhorando, em certos aspectos, as coisas tão voltando ao normal (sd2)As pessoas tão criando mais humildade umas com as outra.* tão sabendo...(sd3) tão sabendo ter Deus mais no coração...(sd4) *dar valor nas coisas que perde...(sd5).* (SD4).

Recorte 7: *Ah a gente imaginava que as pessoas fossem mudar um pouco, que as pessoas fossem parar e pisar um pouco no freio (sd4), e valorizar um pouco mais a vida como as pessoas romantizaram tanto durante a pandemia (sd5), mas agora que voltou tudo ao normal todo mundo esqueceu né (sd6), essa visão romantizada e infelizmente a humanidade caminha no seu normal de novo (sd7).* (SD12).

Os recortes de 8 a 11 enunciam efeitos de sentido afetados pelas memórias discursivas advindas do modelo de doença. Os discursos foram afetados pelo paradigma biomédico e o modelo problema-solução, ideias pré-construídas e consolidadas de maneira hegemônica na história da saúde e do cuidado.

Recorte 8: *Sobre a pandemia, ela piorou muito o meu estado (sd11). Em primeiro lugar, eu espero a minha cura (sd12). E sei que em nome de Jesus, eu vou me curar (sd13). Eu vou conseguir me libertar de todos esses remédios, porque eles não são brincadeira (sd14).* (SD7).

Recorte 9: *Eu espero não ter recaídas (sd6). Recaídas a gente pode ter né, mas eu tô reagindo contra (sd7).* (SD5).

Recorte 10: *[Na pandemia] eu traficava, então eu tinha droga dentro de casa do mesmo jeito, aí que eu usava mais ainda (sd1). Não tinha nada pra fazer, aí eu fervia usando mais ainda (sd2). [Hoje tenho] Sentimento de que eu tenho que viver no remédio* né...porque *senão, não guenta (sd3).* (SD9).

Recorte 11: *Aí descobri que tava com depressão, agora a depressão está instalada (sd6), ela [psicóloga da unidade] já me falou que tá instalada, então tenho que fazer de tudo pra sair (sd7), então é como uma droga, você vai, um dia cê tá bem, outro dia cê não tá bem, tá? (sd8) [...] eu preciso sair dessa depressão que eu tô, pra da outra vez falar pra você que eu tenho sonhos, porque eu não tenho mais (sd10). Esperança pra mim significava muita coisa sabe, esperança pra mim era tudo, era uma palavra-chave (sd12)[...]agora filha, vou falar pra você, na depressão que eu tô, como tá instalada, se eu falar pra você que eu tenho objetivo na vida, eu vou tá mentindo (sd14).* (SD13).

Os recortes de 12 a 15 expressam sentidos de esperança advindos de memórias discursivas ancorados no discurso de resiliência. Os recortes enunciam sentidos de valorização de recursos internos e pessoais para a superação de adversidades. Alguns discursos reiteram a ideia de que as dificuldades são importantes para trazer força interna.

Recorte 12: *Ela [a pandemia] tá sendo assim, basicamente, de aprendizado, sabe? (sd7) foi assim, no sentido de... Sentido de olhar as coisas sabe? (sd8) que a pandemia pra mim, ela não foi fácil (sd9). Ela foi uma tremenda bagunça (sd10). Então, ela foi bem... é... teve um desequilíbrio da minha saúde mental (sd11) [...] mas aí eu acabei levantando e... fui mudando um pouco a situação(sd12). [Minha esperança] vem mais mesmo da minha coragem, digamos assim. Às vezes, eu tenho uma coragem mesmo de viver mais a vida. Além de desanimar bastante, como às vezes acontece, é... às vezes, eu sou bastante corajosa em fazer algumas coisas (sd13).* (SD1).

Recorte 13: *não foi fácil, pra ninguém foi fácil, todo mundo sofreu, as consequências da pandemia (sd3), então nós somos sobreviventes de um naufrágio...(sd4) estamos sobrevivendo ainda, graças a Deus...(sd5).* (SD14).

Recorte 14: *Então, eu espero felicidade, correr atrás de todos os meus objetivos (sd12). Acordar cedo e ir para a luta, ter uma vida normal, como qualquer outra pessoa tem (sd13).* (SD5).

Recorte 15: *[Esperança significa] Tentar alcançar os pontos positivos na vida... (sd9)“ah! eu tenho esperança de não ter problema”...(sd10) mas tem que ter problema na vida pra crescer...(sd11) tiro [esperança] de mim mesmo e um pouquinho da fé que eu tenho, em Deus ... (sd12) é que minha vida foi meio complicada desde criança, então acho que foi eu mesmo, aí precisei ter mais força pra eu poder lutar (sd13).* (SD6).

## DISCUSSÃO

O estudo apontou que os sujeitos produziram diversos sentidos sobre esperança no período pandêmico, sentidos estes afetados pelas memórias discursivas, as quais não são acessíveis conscientemente pelo sujeito, mas são constitutivas dos discursos^(12,13)^. Os sentidos já estão no mundo antes, no já-dito, no pré-construído e retornam no dizer^(15)^.

Os recortes de 1 a 4 expressam sentidos de esperança permeados pelas memórias discursivas construídas pelo discurso religioso/de espiritualidade. Religiosidade pode ser definida como as ações, práticas e crenças articuladas a determinada religião. A espiritualidade possui sentido mais abrangente, envolvendo a busca pessoal por questões essenciais à vida e conexão com o sagrado ou transcendental^(16)^. O recorte 1 apresenta a resposta da SD5 à pergunta - “Como você descreveria a esperança na sua vida?”. As condições de produção dos discursos são de uma usuária do serviço CAPS AD, que está retornando ao tratamento há pouco tempo e sofre as consequências do uso abusivo de SPA. O recorte 1 enuncia que o sentido de esperança é a ausência das drogas. Na sd8 a expressão “as coisas que eu fazia” silencia o sentido “usar drogas”, sendo que, na perspectiva de SD5, tal consumo é considerado inconveniente ou inadequado. Esse silêncio consiste na ocultação de outros sentidos possíveis que causam desconforto, ou seja, concebido como inconveniente^(13)^.

Em efeito metafórico de deslizamento de sentidos “mundo escuro” significa “drogas” e a droga cega a pessoa e tudo é impossível, enquanto que o sentido oposto, a “luz” seria a figura de Deus, ou seja, quando ela sai da escuridão, “enxerga” e “vê” a existência de Deus. Nesse sentido, as drogas são a escuridão e Deus é a luz. O paradoxo entre escuridão e luz remete ao discurso religioso de que a figura de Deus seria a solução para a problemática do uso de SPA. Como a SD5 enuncia, quando ela enxerga Deus, “tudo é possível” (sd9) e “tudo pode dar certo”(sd10).

No recorte 2, o sentido de esperança é personificado na figura de Jesus. Em deslocamento de sentidos, o significante “esperança” ganha um sentido espiritual e religioso. O seguimento discursivo - “fazer a coisa certa” - silencia outros sentidos possíveis de que a coisa certa seria a crença em Jesus e a vontade, o desejo em ter fé. Esse recorte expressou indícios de memórias discursivas articuladas ao discurso religioso e de justiça/ética, em que a esperança pertence ao universo da fé, do transcendente, mas da justiça/ética, ou seja, de fazer a coisa certa.

O Recorte 3 apresenta a resposta da SD5 à pergunta - “Como você descreveria a esperança na sua vida?” O sentido de esperança ganha o sentido de que Deus ajuda as pessoas a não perderem a esperança. A ideia de esperançar uma nova vida, um novo recomeço (perspectiva futura) – emerge na paráfrase “Nunca é tarde para recomeçar” (sd14). A sd11 reforça o discurso de espiritualidade por meio da paráfrase “Deus é amor nas nossas vida”. O pré-construído em outro lugar retorna no dizer demonstrando sentidos de que a espiritualidade expressa sentidos de amor, caridade, ajuda e esperança.

A SD14 enuncia que a esperança em sua vida seria compartilhar esse sentido com outras pessoas. O sujeito elenca ações de cunho espiritual que para ela se relacionam com o sentido de esperança (orar, interceder, falar de Deus para as pessoas). Essas ações fazem parte da dimensão comportamental da esperança do Modelo de Esperança, a qual envolve as ações de caráter social, psicológico, físico ou religioso implicadas no processo de esperar e ter esperança^(9)^.

O Recorte 4 apresenta a resposta da SD5 à pergunta: “Como você descreveria a esperança na sua vida?”. Em efeito metafórico, o sentido de esperança é deslocado para o sentido de fé, em movimento de transferência contextual. Fé e esperança, para a SD6 são “quase” como sinônimos, ou seja, para ela o sentido de esperança está intimamente conectado com a crença na espiritualidade. Parafraseando o modelo de Esperança de Dufault e Martocchio^(9)^, há um sentido de esperança generalizada, ou seja, não referente a um objeto de esperança específico, mas sim de sentimento generalizado de um “amanhã melhor”, de um “futuro melhor” e na expectativa por mudanças no presente.

As produções discursivas religiosas/de espiritualidade exemplificam como a dimensão afiliativa do processo de esperança é mobilizada^(9)^, a qual se refere às relações com outras pessoas (vivas ou mortas) ou com um Ser Superior. Os recortes discursivos enunciam que relacionar-se com Deus constitui-se em fonte de esperança para os sujeitos do discurso. A espiritualidade insere-se na dimensão comportamental da esperança^(9)^, considerando-se que ritos e práticas religiosas, tais como orações, meditações, cultos e participação em instituições religiosas, por exemplo, são ações e comportamentos que podem facilitar o surgimento ou fortalecimento do estado de esperança.

Estudos^(17,18)^ corroboram o pressuposto de que a dimensão da espiritualidade está intimamente relacionada com a experiência da esperança e demonstram que as crenças espirituais são recursos internos significativos para a obtenção ou manutenção de esperança. A espiritualidade foi identificada como fonte de esperança nestes estudos^(17,18)^.

Os discursos acima evocam memórias discursivas advindas do discurso religioso/de espiritualidade. Os sentidos construídos sobre esperança são fortemente atravessados pelos sentidos de fé, crença e espiritualidade. Como condição de produção ampla, destaca-se que a população brasileira é em sua maioria cristã, de religiões católica ou evangélica^(19)^. As condições de produção referem-se às circunstâncias em que os discursos são produzidos e sua relação para permitir o dizer. Envolvem o contexto sócio-histórico, aspectos imaginários e ideológicos em que o sujeito está inserido^(13)^.

A religião apresenta-se como uma forma própria de linguagem que possui seus códigos e ritos e atua na mediação do processo de significação da vida e da realidade^(16)^. As religiões, enquanto práticas sociais, fazem parte da constituição do sujeito e organização social, influenciando o modo de ser e construir sentidos dos sujeitos^(16,20)^, portanto é esperado que a espiritualidade seja transversal aos discursos dos sujeitos discursivos deste estudo.

Outro discurso evidenciado neste estudo envolveu o apelo à solidariedade como forma de lidar com as dificuldades impostas pela pandemia. A disseminação veiculada pelas redes sociais e discursos midiáticos foi de que a pandemia proporcionou sensação positiva, ou seja, a pandemia poderia invocar a experiência e sentimentos solidários nas pessoas, bem como a percepção de que a adversidade pandêmica desencadearia sentimentos de valorizar mais a vida das pessoas.

O discurso de solidariedade é retomado por meio de processos parafrásicos nos recortes discursivos de 5 a 8. O Recorte 5 é parte da resposta dada pela SD1 à pergunta: “Quando você escuta a palavra esperança durante a pandemia, o que te vem à cabeça?”. Em efeito metafórico, a SD1 resgata a memória discursiva de que a cor verde simboliza a esperança. A cor verde, no imaginário social relaciona-se à natureza, germinação e renascimento; preservar o ambiente, o equilíbrio e a harmonia (contrário ao ambiente de briga e discordância). Os seguimentos discursivos “assim, de solidariedade... de mais afeto, mais carinho... (sd4) as pessoas... elas deveriam ser mais assim (sd5). Hoje em dia... hoje em dia tem muita briga, muita discordância... (sd6)” revelam indícios de sentido de esperança relacionados com o sentido de solidariedade. Há a relação de efeitos de sentido ancorados no Interdiscurso de valorização da solidariedade, ou seja, ser solidário envolve ser afetuoso e carinhoso, bem como manter ambiente harmônico. O sujeito atribui o sentido de solidariedade ao significante “esperança”.

O recorte 6 também enuncia sentidos ancorados no Interdiscurso de solidariedade, em que a pandemia transforma as pessoas mais solidárias e valorizam mais as coisas que perdem. Para o SD4, a esperança no período pandêmico seria ter mais “humildade” entre as pessoas. Percebe-se ato falho quando SD4 escolhe o significante “humildade” e não outros possíveis, como “humanidade ou solidariedade”. Entretanto, no recorte discursivo, o significante “humildade” adquire outro significado quando inscrito nesta determinada formação discursiva - o sentido de solidariedade, sentido este diferente de seu significado literal que, de acordo com o dicionário Aurélio, significa “modéstia, simplicidade”. Para a AD, esse efeito de sentido demonstra como as palavras não possuem significado literal, originário; o sentido pode ser deslocado e receber outro efeito, ou seja, circular entre regiões discursivas diferentes^(21)^.

A solidariedade surge como resposta da sociedade ao período pandêmico, a qual constitui recurso que pode ser mobilizado de maneira individual e/ou coletiva, por empresas, grupos organizados pela sociedade civil, movimentos sociais e pelo poder público^(22)^. No estudo, o sentido de “solidariedade” relaciona-se ao sentido de “solidariedade filantrópica”, expressa pela conduta individual ou de pequenos grupos para realizar ações voluntárias, assistencialistas e caritativas, bem como manter postura empática.

O significante “esperança”, quando pertence ao universo semântico do significante “solidariedade”, remete à dimensão afiliativa do Modelo de Esperança^(9)^. Ajuda mútua, reciprocidade e preocupação com o outro fazem parte do processo de esperança à medida que nos relacionamos uns com os outros e podemos fortalecer a esperança por meio das relações.

O recorte 7 expressa o sentido de que a pandemia desencadearia o bem para a humanidade, ou seja, momentos reflexivos para que as pessoas parassem e levassem a vida “mais devagar”. Segundo SD12 houve uma romantização no período da pandemia, isto é, vivenciar tempos difíceis pode incentivar as pessoas a valorizarem mais a vida. Em efeito de deslizamento de sentidos, no sd4 “as pessoas fossem parar e pisar um pouco no freio” dá efeitos de desaceleração, calma, pausa, o que, na conjuntura dada, implicaria na valorização da vida (sd5). Entretanto, no sd6, a SD12 conclui que não houve essa mudança esperada, ao “voltar tudo ao normal”, a “humanidade caminha no seu normal”, ou seja, retorna ao ritmo acelerado, ritmo este que não proporciona a valorização da vida. Outra pista importante neste recorte discursivo é a “visão romantizada” mencionada no sd7, que remete às memórias discursivas disseminadas na pandemia, de um período bom para a humanidade, ou seja, de valorizar a vida diante das adversidades pandêmicas.

Durante o período pandêmico, as redes sociais online e midiáticas expuseram o imaginário social diversificado, ou seja, parte da população privilegiou disseminar informações sobre a pandemia, outra parcela enfatizou sobre o potencial perigoso do vírus e as perdas ocasionadas por complicações da COVID-19, e outra expressou experiências positivas advindas da pandemia, como por exemplo relatos de solidariedade e atos caridosos, experiências individuais de superação e experiências pessoais positivas de reinvenções de hábitos e rotinas, conforme literatura^(23)^.

Os recortes de 8 a 11 enunciam efeitos de sentido afetados pelas memórias discursivas advindas do modelo de doença, em que os sintomas e as medicações adquirem posições centrais no processo terapêutico, baseando-se na premissa de “doença-cura” e “problema-solução”. No âmbito do uso de álcool e outras drogas, a dependência química é doença e a sua “cura” é somente por meio da abstemia^(24)^. No contexto de transtornos mentais e os derivados de consumo de SPA, o transtorno mental é uma doença que pode ser tratada por meio de medicações, constituindo-se um problema que tem uma causa e requer uma solução, neste caso, o medicamento.

O paradigma racionalista de “problema-solução” era hegemônico até as primeiras experiências de Reforma Psiquiátrica no período de 1960 e subsidiava as práticas em Saúde Mental e Psiquiatria. Nesse paradigma, o problema dado seria a doença e a solução esperada seria a cura. A terapia nessa lógica é considerada um processo que articula o diagnóstico ao prognóstico esperado – a cura. Com as primeiras iniciativas de Reforma e o avanço do conhecimento na área de saúde mental, entende-se que as pessoas com transtornos mentais possuem uma experiência integral, complexa e concreta que não pode ser reduzida à lógica “problema-solução”^(25)^. Para atuar na prática com usuários de serviços especializados de saúde mental é necessário superar o paradigma problema-solução e caminhar para um sentido de que, no âmbito dos transtornos mentais, o sofrimento psíquico é uma experiência subjetiva e complexa em contexto social e, no âmbito do consumo de álcool e drogas, existem abordagens que reconhecem a autonomia, protagonismo e singularidade dos usuários, como por exemplo a abordagem da Redução de Danos^(25,26)^. Dessa forma, consideram-se os usuários sujeitos sociais e atores da mudança e de reconstituição de sentidos de vida^(25)^.

No recorte 9, a SD5 tem esperança em não ter mais “recaídas”, sendo que este significante revela indícios de um discurso fundamentado no modelo de doença e de prevenção de recaídas. Nesta perspectiva, a dependência de SPA é uma doença e, portanto, busca-se atingir a cura, ou seja, a abstemia. Recair significa voltar ao uso, o qual deve ser prevenido neste modelo^(24)^.

Os recortes acima expressam a dimensão cognitiva do processo de esperar e ter esperança, quando os sujeitos discursivos elaboram, interpretam, julgam e elencam seus objetos de esperança, definindo prioridades, por exemplo^(9)^. A SD2 atribui o significado de esperança ao desejo de “recuperação” e SD7 destaca a cura como seu principal e primeiro objeto de esperança. A SD5 define como seu objeto de esperança a abstemia das SPA, mantendo-se livre de recaídas.

No recorte 10, o sd1 aponta contradição e crítica diante da posição do sujeito discursivo em sua posição social, em um determinado momento de sua vida, ou seja, de ser traficante e consumidor de SPA, ao mesmo tempo. O significante “fervia” na sd2 denota sentidos de ebulição, efervescência, borbulhar o que enuncia sentidos de uso de drogas intenso, principalmente na pandemia. Destaca-se em sd3 - “eu tenho que viver no remédio” - o enunciado de sentidos retoma as memórias discursivas do modelo de doença, em que as medicações são a principal solução terapêutica para o sofrimento psíquico^(24)^. O discurso salienta não só a dependência da SPA, mas também dos fármacos, o que sustenta o gerir da vida do enunciador.

No recorte 11, a SD13 refere não ter esperança devido à depressão “instalada”, sendo este significante em efeito metafórico de transferência contextual, ou seja, sentidos de “concretizada”, “assentada”, “diagnosticada”. Para a SD13 o fato de seu transtorno mental estar “instalado” condiciona e determina sua experiência de esperança. As marcas linguísticas e os vestígios derivam efeitos metafóricos de que a usuária teria que “fazer de tudo pra sair” da depressão, curar-se, para que em tempo futuro, “da outra vez”, pudesse ter sonhos e objetivos.

A condição do diagnóstico de depressão, ou ausência deste, é determinante para a capacidade de ter esperança. A dimensão contextual influencia no processo de esperança, pois as circunstâncias e situações da vida interferem na capacidade do sujeito de manter a esperança^(9)^. A condição de produção estrita de agravamento da depressão e a vivência de situação de luto de SD13 devido à pandemia, desencadeou-lhe sentimentos de desesperança.

O pressuposto de que desesperança e esperança não são antagônicas reforça a percepção de que uma mesma situação pode provocar oportunidades para a esperança ou desesperança, dependendo de como o sujeito percebe, interpreta e reage ao contexto. Contextos que denotam desesperança podem despertar para novas metas, revisar planos e suas estratégias^(9)^.

Os recortes de 12 a 15 enunciam sentidos de esperança que são afetados por memórias discursivas pré-construídas, do discurso de resiliência. A SD1 relata “desequilíbrio da saúde mental” quando define o significado pessoal de esperança em tempos pandêmicos. O significante “desequilíbrio” denota deriva de efeitos metafóricos de sentidos de que o sofrimento psíquico a faz perder o equilíbrio e cair, apesar do sujeito discursivo reconhecer sua “coragem” como fonte de esperança motivadora para “se levantar” e “mudar a situação”. Esses sentidos apontam para a valorização de características internas e recursos pessoais de enfrentamento e resiliência, buscando superar as dificuldades do período pandêmico.

Resgatando as condições de produção estritas de SD1 em que o discurso foi enunciado, destaca-se a linguagem não verbal da entrevistada, expressa pela sua postura cansada e desanimada. Percebe-se contradição e ambivalência diante do reconhecimento da “coragem” de SD1 e a posição-sujeito de desânimo. A esperança está intimamente articulada à condição de incerteza e é caracterizada como experiência humana que pode oscilar, alternando entre momentos de alta e baixa esperança. Algumas respostas afetivas para as incertezas são sentimentos de ansiedade, vulnerabilidade, preocupação e tristeza das pessoas^(9)^.

No recorte discursivo 13, a SD14 utiliza-se da figura de linguagem metáfora para comparar a sociedade com um navio afundando no período da pandemia, denotando sentidos de destruição e aniquilamento, mas com sobreviventes. O sujeito discursivo salienta a empatia, ao enunciar o sofrimento compartilhado e vivenciado coletivamente neste período pelas pessoas. A escolha pelo significante “sobreviventes” por sua vez sugere sentidos de resiliência. Na condição de produção estrita em que o discurso foi emitido, a SD14 acredita que é possível se adaptar e superar as dificuldades impostas pela pandemia, ou seja, “sobreviver ao naufrágio”.

No recorte 14 o sujeito do discurso atribui ao significante “esperança” sentidos de resiliência e esforço elucidando o funcionamento da dimensão comportamental da esperança, que envolve as ações mobilizadas pelos sujeitos em alcançar algo significativo para si e para os outros^(9)^. O sujeito discursivo elabora estratégias relacionadas ao campo da ação, do agir (acordar cedo, correr atrás, ir à luta), as quais a ajudarão a atingir seu resultado esperado. A sd13 enuncia a marca linguística da paráfrase, quando SD5, no seu dizer - “Acordar cedo e ir para a luta” - parafraseia o ditado popular - “Deus ajuda quem cedo madruga”. O mecanismo de paráfrase retoma sentidos pré-construídos de valorização da dedicação e esforço.

Para a SD5, tais sentidos são atribuídos a “ter uma vida normal”, ao escolher o significante “normal” e não outros. Esse fenômeno da AD é chamado de esquecimento n.2, em que o sujeito do discurso privilegia e seleciona determinados dizeres em detrimento de outros^(12)^. A escolha em dizer determinada palavra e não outra possui um significado que está relacionado com as condições de produção amplas e estritas, com o histórico e o ideológico^(13)^. O “normal” para a SD5 relaciona-se ao trabalho duro, funcionalidade e luta.

Percebe-se que o sentido de resiliência é transversal aos recortes acima, o qual é compreendido enquanto fenômeno complexo, que vem sendo estudado ao longo dos últimos anos por diversas áreas, tais como Ciências Humanas e Ciências da Saúde e conceituado por diversos autores^(27,28)^. A resiliência pode ser definida como processo de enfrentamento a desafios ou adversidades e como a capacidade de elaborar respostas adaptativas para obter resultados esperados. É um processo desenvolvido ao longo da vida que envolve recursos de enfrentamento pessoais, interpessoais e sociais^(29)^. O sentido de resiliência, pré-construído anteriormente e em outro lugar, é retomado pelos sujeitos e materializado em seus discursos, quando estes descrevem sua experiência de esperança durante a pandemia da COVID-19.

No recorte 15 o sentido desvelado é o de valorizar a superação das adversidades, durante a resposta à pergunta: “O que significa esperança pra você?” O sentido evidenciado é o de resiliência e superação, pois o sujeito mobiliza recursos internos (força pessoal/resiliência) e recursos externos (um pouquinho de fé) para empoderar-se perante as adversidades. O sd11- “tem que ter problema na vida pra crescer” - apresenta a pista linguística de paráfrase, compreendida enquanto retorno da memória no dizer^(13)^. Essa sequência discursiva retoma sentidos pré-construídos em outro lugar de que - “o sofrimento faz as pessoas crescerem” e de que as dificuldades possibilitam aprendizados e mudanças positivas.

Para a AD, ao analisar a constituição de um discurso, analisam-se os discursos que nele se inserem, ou seja, o Interdiscurso^(30)^. A sd11 retoma o discurso socialmente disseminado, portanto, de formações ideológicas de valorizar as adversidades. Para o sujeito, a esperança adquire o sentido de atravessar as dificuldades da vida e a sd13 -”precisei ter mais força pra eu poder lutar” - reforça a valorização do esforço individual, da habilidade e competência para enfrentar as adversidades.

Para a AD, as condições de produção referem-se a uma soma de variáveis que compõe a situação em que o discurso está sendo emitido, considerando o contexto sócio-histórico, contexto pessoal e o lugar de onde o sujeito fala^(30)^. Neste recorte percebe-se que diante de um contexto macro (pandemia) e contexto micro (história de vida pessoal), o sujeito vivenciou situações de vida difíceis - “minha vida foi meio complicada desde criança” - impulsionando-o a “lutar” contra as adversidades.

Interpreta-se que a capacidade de aprendizado diante do enfrentamento do problema no passado ou no presente subsidia um futuro de resolução do problema, mas não a exclusão deste no passado, presente e futuro. Portanto, o objetivo consiste em esperançar competência e habilidade para resolver problemas futuros. Assim, tal contexto envolve a dimensão temporal da esperança, em que a vivência do passado e presente subsidia o futuro, para possibilitar a competência e habilidade^(9)^.

No referente às limitações deste estudo, podem estar relacionadas ao período da coleta de dados concernente ao momento da onda pandêmica da COVID-19, considerando-se as condições de produção estrita em que a vacinação foi iniciada e o término desta doença, enquanto emergência de saúde pública. Outra limitação corresponde ao predomínio do gênero feminino dos participantes do estudo em relação à percepção do sentido de esperança, requerendo ampliação do estudo com a população de gênero masculino.

Os resultados deste estudo contribuem para uma escuta mais empática e sensível à experiência da esperança advinda dos discursos de usuários de serviços especializados em saúde mental durante o período pandêmico da COVID-19, o que pode favorecer tanto ao profissional de saúde, principalmente o(a) enfermeiro(a), como aos usuários do serviço de saúde mental, a (re)conhecer a esperança de si e do outro como fontes promotoras de saúde mental.

## CONCLUSÃO

Os discursos produzidos sobre a esperança evocam memórias discursivas construídas anterior e exteriormente ao sujeito. Os sentidos da esperança foram perpassados pelos discursos: religioso/de espiritualidade, de solidariedade, do modelo de doença e de resiliência. Acredita-se ser importante capacitar os profissionais de saúde, principalmente enfermeiros(as), que atuam nos CAPS e CAPS AD, a apreenderem os sentidos construídos da esperança e a reconhecerem-nos enquanto recurso terapêutico constituinte do cuidado. Destaca-se a importância em reconhecer a promoção de esperança enquanto intervenção na prática clínica e de cuidado.

# Translation: Sentidos de esperanza construidos por usuarios de servicios de salud mental durante la pandemia de COVID-19

**DOI:** 10.1590/1980-220X-REEUSP-2024-0182es

**Authors:** Sarah Salvador Pereira, Angélica Martins de Souza Gonçalves, Simone Teresinha Protti-Zanatta, Maria do Perpétuo Socorro de Sousa Nóbrega, Bianca Cristina Ciccone Giacon, Sonia Regina Zerbetto

**Affiliations:** 1 Universidade Federal de São Carlos, Departamento de Enfermagem, Programa de Pós-graduação em Enfermagem, São Carlos, SP, Brasil.; 2 Universidade de São Paulo, Escola de Enfermagem, Programa de Pós-graduação em Enfermagem, São Paulo, SP, Brasil.; 3 Universidade Federal de Mato Grosso do Sul, Instituto Integrado em Saúde, Programa de Pós-graduação em Enfermagem, Campo Grande, MS, Brasil.

**Keywords:** Salud Mental, Esperanza, COVID-19

## Abstract

**Objetivo:**

Analizar la producción de sentidos de esperanza en los discursos de usuarios de servicios especializados de salud mental de una ciudad del interior de Minas Gerais en el contexto de la pandemia de COVID-19.

**Método:**

Estudio cualitativo basado en el marco teórico-metodológico del Análisis del Discurso francés y el constructo teórico de la esperanza. Se realizaron entrevistas semiestructuradas de agosto de 2022 a mayo de 2023.

**Resultados:**

El bloque discursivo titulado “Los sentidos de la Esperanza construidos en la pandemia del COVID-19” aborda los sentidos construidos sobre la esperanza que se encuentran permeados por los efectos que surgen de las memorias discursivas. Los extractos discursivos plantean indicios del discurso religioso/espiritual; discurso de solidaridad; modelo de enfermedad; y el discurso de la resiliencia.

**Conclusión:**

Los discursos preconstruidos en otros lugares sobre espiritualidad/religiosidad, solidaridad, modelo de enfermedad y resiliencia permean la producción de significados de esperanza en los discursos de los usuarios de servicios de salud mental. Es importante permitir que los enfermeros comprendan los significados construidos de la esperanza y los reconozcan como un recurso terapéutico que constituye el cuidado.

## INTRODUCCIÓN

La pandemia de COVID-19 (Enfermedad por Coronavirus 2019), como emergencia de salud pública internacional, fue declarada en 2020 por la Organización Mundial de la Salud (OMS) y ha desencadenado no sólo retos para los sistemas sanitarios y las políticas de salud pública en todo el mundo^(1)^, sino también cambios en todos los sectores de la sociedad, afectando a las condiciones de trabajo/empleo, educación y especialmente a la salud mental de la población.

En la literatura se han descrito síntomas como insomnio, estrés, ansiedad y síntomas depresivos en la población en situaciones pandémicas^(2)^. Durante la pandemia de COVID-19, la población ha experimentado diversas dificultades que han afectado a su salud mental, tales como: miedo a esta enfermedad y a la muerte; miedo relacionado con la disminución de la fuente de ingresos; alteraciones del sueño; sentimientos de desesperanza, soledad y síntomas depresivos; ira, frustración o irritabilidad; miedo a la posibilidad de que el individuo o miembros de su familia contraigan COVID-19, ansiedad u otras reacciones de estrés^(3)^.

Toda la sociedad ha sufrido y sigue sufriendo las consecuencias de la pandemia del COVID-19; sin embargo, algunos segmentos de la población han estado más expuestos a los riesgos del COVID-19 y se han encontrado más vulnerables, como las personas que padecen trastornos mentales o trastornos relacionados con el consumo de sustancias psicoactivas (SPA)^(3,4)^. En cuanto a las personas que consumen SPA, el periodo pandémico ha influido e intensificado sus condiciones clínicas y psicosociales, incluyendo un aumento de este consumo^(5)^. Además, la adherencia de estos usuarios al tratamiento en los servicios sanitarios se ha visto comprometida debido, por ejemplo, a los cambios sociales y económicos^(6,7,8)^.

Se supone que durante la pandemia de COVID-19, la población necesitaba esperanza para afrontar los retos diarios. Se cree que la esperanza se ha visto aún más comprometida en la percepción de los usuarios de los servicios especializados de salud mental, debido a factores como aislamiento social, cambios en la rutina diaria, dificultad de acceso a la atención ofrecida por los servicios de salud mental, cambios en el ambiente de trabajo, dudas e incertidumbres sobre la enfermedad COVID-19^(3)^.

Frente a este escenario de adversidades y desafíos, tener y mantener la esperanza puede ser un recurso importante para promover la salud mental y motivar a las personas a (re)construir y conducir sus vidas. El significado de esperanza adoptado en este estudio es el de una fuerza vital, dinámica, compleja y multidimensional. Es un proceso caracterizado por la expectativa confiada de alcanzar un objetivo futuro significativo para la persona^(9)^. Es un recurso psicosocial que promueve condiciones para enfrentar situaciones de crisis y tiene efectos favorables sobre la salud. Es una herramienta de apoyo para afrontar los problemas relacionados con las necesidades de salud y mantener la calidad de vida^(10)^.

Por todo lo expuesto, se constata la relevancia científica y social del tema de la esperanza en salud mental durante la pandemia del COVID-19, especialmente en el contexto del alcohol y otras drogas, y nos preguntamos: ¿cuáles son los significados de esperanza construidos por los usuarios de los servicios especializados de salud mental en el contexto de la pandemia del COVID-19?

El objetivo de este estudio fue analizar la producción de significados de esperanza en los discursos de los usuarios de los servicios especializados de salud mental de una ciudad del interior de Minas Gerais en el contexto de la pandemia del COVID-19.

## MÉTODO

### Tipo

Estudio cualitativo, exploratorio, guiado por la herramienta*Consolidated Criteria for Reporting Qualitative Research*(COREQ). El estudio se basó en el marco teórico y metodológico del Análisis del Discurso (AD) de matriz francesa de Michel Pêcheux y su afiliada Eni Orlandi, que introdujeron el marco en Brasil, y en el marco teórico interpretativo del Modelo de Esperanza de Dufault y Martocchio.

El Análisis del Discurso se ocupa de los efectos de los significados producidos entre los hablantes en sus ubicaciones sociales determinadas por la estructura social^(11)^. Para el AD, los significados se construyen socialmente y están determinados por la historia, la ideología y el inconsciente^(12)^. La AD permite interpretar el discurso identificando las huellas y marcas del contexto ideológico, histórico y social, que no son organizadas por el propio enunciador, sino que se revelan en la materialidad lingüística^(13)^.

Los significados producidos por los sujetos se imprimen en Formaciones Discursivas (FD), que constituyen algo que, en una posición y situación dadas, permite lo que puede y debe ser dicho. Por lo tanto, una determinada DF producida por el sujeto es interpelada por otras DF construidas antes y fuera de él. En esta configuración, encontramos el olvido número 1, término utilizado en AD para designar la ilusión de que somos los originadores del pensamiento o del decir^(13)^.

El modelo de Esperanza de Dufault y Martocchio^(9)^sostiene que la esperanza puede definirse como una fuerza vital dinámica que implica varias dimensiones caracterizadas por la expectativa confiada de alcanzar un objetivo futuro significativo para la persona. Las seis dimensiones de la esperanza son: afectiva, cognitiva, conductual, contextual, afiliativa y temporal^(9)^.

### Localización

La investigación involucró cuatro servicios especializados en salud mental: el Centro de Atención Psicosocial al Alcohol y Drogas (CAPS AD), el Centro de Atención Psicosocial II, el Centro Multiprofesional de Salud Mental y el Centro de Atención a la Salud Mental Post-Covid, pertenecientes a una ciudad del interior de Minas Gerais.

### Población y Criterios de Selección

Se utilizó una muestra de conveniencia de 14 usuarios de estos servicios especializados en salud mental. Los criterios de inclusión fueron: tener 18 años o más y estar en tratamiento en el servicio especializado de salud mental; y los criterios de exclusión fueron tener dificultad de comprensión, lo que impidió la realización de las entrevistas y/o estar intoxicado o en brote psicótico el día de la entrevista, presentando signos físicos y psicológicos alterados. En la intoxicación, según el PAS, serían: labilidad intensa del humor, euforia, somnolencia, incoordinación motora y confusión mental. En un brote psicótico, serían: agitación, agresividad, confusión mental, alucinaciones o delirios.

La muestra se cerró por saturación teórica de datos, donde se considera que el punto de saturación es cuando se entiende que se han obtenido datos suficientes para comprender al sujeto^(14)^.

### Recogida de Datos

Los datos fueron recogidos desde agosto de 2022 hasta mayo de 2023 por la investigadora principal del estudio (estudiante de doctorado), con experiencia en salud mental, en el tema de la esperanza y en entrevistas para investigación cualitativa. Se utilizó un cuestionario sociodemográfico y clínico para caracterizar a los participantes en el estudio (con preguntas sobre género, religión, educación, estado civil, situación laboral actual, duración del tratamiento en el servicio y satisfacción con el tratamiento) y un guión de entrevista semiestructurada con preguntas que indagaban sobre el significado de la esperanza para los usuarios durante la pandemia de COVID-19, perspectivas futuras en sus vidas, factores que inhiben y facilitan la esperanza. Las entrevistas duraron una media de 25 minutos y fueron grabadas en audio.

Los participantes fueron reclutados mediante contacto previo y derivación por los profesionales sanitarios de los respectivos servicios, previa autorización de los usuarios, o por el investigador en la sala de espera. Las entrevistas tuvieron lugar en estos servicios, en una sala privada, garantizando la privacidad y respetando los protocolos sanitarios y las medidas de seguridad frente al COVID-19 determinadas en su momento por el Ministerio de Sanidad.

Los participantes fueron identificados como Sujetos Discursivos (SD) seguidos de una numeración secuencial en el orden de las entrevistas. Las secuencias discursivas se representaron con la sigla «DS», seguida de la numeración, y se subrayaron los textos con trazos y marcas lingüísticas.

### Análisis y Tratamiento de Los Datos

Se utilizaron los dispositivos analíticos del marco metodológico AD^(13)^, que permitieron captar lo dicho y lo no dicho más allá de la superficie y analizar la materialidad que vehicula el discurso. La primera etapa consistió en transcribir las entrevistas, identificando huellas y marcas lingüísticas para comprender cómo funciona el discurso. La segunda etapa nos permitió comprender cómo se constituyen los significados en un discurso determinado, identificando segmentos y secciones discursivas. En la siguiente etapa, los extractos discursivos se agruparon en bloques discursivos según formaciones ideológicas similares. Para validar y garantizar la fiabilidad de las secuencias discursivas, los extractos y los bloques discursivos, participaron tres enfermeras investigadoras: una sobre el tema de la esperanza y la salud mental, otra sobre el tema del alcohol y otras drogas, y una tercera sobre la EA.

### Aspectos Éticos

Este estudio fue aprobado por el Comité de Ética para la Investigación con Seres Humanos de una Universidad Federal en 2022 bajo el dictamen nº 5.264316. El estudio cumple con la Resolución 510/16 del Consejo Nacional de Salud. Los participantes firmaron el consentimiento informado.

## RESULTADOS

Un total de 18 usuarios del servicio fueron invitados a participar del estudio, de los cuales 4 declinaron la invitación y no hubo retiros una vez iniciada la entrevista. Se realizaron 14 entrevistas a usuarios de servicios especializados de salud mental: 5 usuarios del Centro Multiprofesional de Salud Mental, 4 usuarios del CAPS II, 3 usuarios del CAPS AD y 2 usuarios del Centro de Atención a la Salud Mental Post-COVID. De estos 14 sujetos, 10 eran mujeres y 4 hombres. La edad media era de 40 años. En cuanto al estado civil, 7 eran solteros, 2 casados, 4 divorciados y 1 viudo; 2 tenían estudios superiores, 5 bachillerato completo, 4 bachillerato incompleto, 1 primaria completa y 2 primaria incompleta. De los usuarios, 7 eran católicos, 4 evangélicos, 1 no tenía religión, 1 era espiritista y 1 agnóstico. Actualmente, 11 personas no trabajaban y 3 sí. El tiempo medio de permanencia en el servicio fue de 18 meses y medio.

El análisis de los datos produjo el bloque discursivo titulado «Los significados de la Esperanza construidos en la pandemia del COVID-19». Este bloque discursivo aborda los significados construidos sobre la esperanza que están impregnados por los efectos de las memorias discursivas. En AD, el interdiscurso se retoma a través de la memoria discursiva, es lo que se ha dicho antes, en otro lugar pre-construido, lo que hace posible el decir. Son las memorias colectivas y sociales que se perfilan en la historia y se materializan en el discurso^(13)^. Los extractos discursivos muestran indicios de un discurso religioso/espiritual, un discurso de solidaridad, un modelo de enfermedad y un discurso de resiliencia.

Los extractos 1 a 4 muestran recuerdos discursivos derivados del discurso religioso/espiritual, en el que las entidades religiosas proporcionan luz, lo que refuerza la existencia de Dios. Existe la perspectiva de un futuro mejor y transformador, apoyado por la fe, la religión y la espiritualidad.

Extracto 1:*[La esperanza] es dejar de hacer las cosas que solía hacer (sd8). No vivir en ese mundo oscuro, ver que todo es posible, que Dios existe (sd9). Y que todo puede salir bien (sd10).*(SD5).

Extracto 2:*Jesús lo es todo (sd6). La esperanza es Jesús (sd7).*¿Te he respondido bien?*La esperanza somos nosotros haciendo lo correcto y queriendo tener fe (sd8).*(SD11).

Extracto 3: *rezar, interceder, hablarle a la gente un poco del amor de Dios, (sd10) Dios es amor en nuestras vidas,(sd11) expresar un poco la parte espiritual para que la gente tampoco pierda la esperanza (sd12) y mostrarles que nada está perdido, cierto, (sd13) nunca es tarde para empezar de nuevo...ya sabes, una nueva vida (sd14).*(SD14).

Extracto 4: *Mi esperanza viene de Dios, viene de Jesús (sd10). Puedo poner fe con esperanza, casi la misma palabra (sd11). Tengo fe en que mañana será mejor, y tengo esperanza en que mañana será mejor, en que todo cambiará (sd12).*(SD6).

Los extractos 5 a 7 muestran discursos apoyados en el Interdiscurso de la solidaridad, expresando la idea de que la pandemia del COVID-19 ha inspirado un sentido de solidaridad en la sociedad, así como afecto, humildad y un sentido de valoración de las cosas que nos rodean.

Extracto 5: *[La esperanza es] un símbolo verde, ¿sabes? (sd3) así, de solidaridad... de más cariño, más cuidado... (sd4) la gente... debería ser más así (sd5). Hoy en día... hoy en día hay mucha pelea, mucho desacuerdo*... (*sd6)*(SD1)*.*

Extracto 6:*Siento... que [la pandemia] ahora está mejorando, en ciertos aspectos, las cosas están volviendo a la normalidad (sd2)La gente se está volviendo más humilde entre sí. Están sabiendo...(sd3) están sabiendo tener más a Dios en sus corazones...(sd4) valorar las cosas que pierden...(sd5).*(SD4).

Extracto 7:*Ah, nos imaginábamos que la gente cambiaría un poco, que la gente se pararía y frenaría un poco (sd4), y valoraría un poco más la vida ya que la gente la romantizó tanto durante la pandemia (sd5), pero ahora que todo ha vuelto a la normalidad todo el mundo ha olvidado, verdad (sd6), esa visión romantizada y desgraciadamente la humanidad ha vuelto a la normalidad (sd7).*(SD12).

Los extractos 8 a 11 enuncian efectos de significado afectados por recuerdos discursivos derivados del modelo de enfermedad. Los discursos fueron afectados por el paradigma biomédico y el modelo problema-solución, ideas pre-construidas y consolidadas hegemónicamente en la historia de la salud y del cuidado.

Extracto 8: *La pandemia ha empeorado mucho mi estado (sd11). En primer lugar, espero mi curación (sd12). Y sé que en el nombre de Jesús me curaré (sd13). Voy a librarme de todos estos medicamentos, porque no son ninguna broma (sd14).*(SD7).

Extracto 9:*Espero no recaer (sd6). Podemos tener recaídas, cierto, pero estoy luchando (sd7).*(SD5).

Extracto 10:*[Durante la pandemia] Yo era traficante de drogas, así que tenía drogas en casa de la misma manera, y luego las consumía aún más (sd1). No tenía nada que hacer, así que me consumía aún más (sd2). [Hoy siento que tengo que vivir de la medicina... porque si no, no lo soporto (sd3).*(SD9).

Extracto 11:*Entonces me enteré de que tenía depresión, ahora está instalada (sd6), ella [la psicóloga de la unidad] me dijo que está instalada, entonces tengo que hacer de todo para salir (sd7), entonces es como una droga, vas, un día estás bien, al otro día no estás bien, ¿no? (sd8) [...] Necesito salir de esta depresión en la que estoy, para que la próxima vez te pueda decir que tengo sueños, porque ya no los tengo (sd10). La esperanza para mí significaba mucho, sabes, la esperanza para mí lo era todo, era una palabra clave (sd12)[...]ahora, hija, te diré que en la depresión en la que estoy, tal y como está montada, si te digo que tengo una meta en la vida, te estoy mintiendo (sd14).*(SD13).

Los extractos 12 a 15 expresan significados de esperanza provenientes de memorias discursivas ancladas en el discurso de la resiliencia. Los extractos enuncian significados de valoración de los recursos internos y personales para superar la adversidad. Algunos de los discursos reiteran la idea de que las dificultades son importantes para sacar a relucir la fuerza interior.

Extracto 12:*Básicamente [la pandemia] es una experiencia de aprendizaje, ¿sabes? (sd7) Fue así, en el sentido de... En el sentido de ver las cosas, ¿sabes? (sd8) la pandemia no fue fácil para mí (sd9). Fue un lío tremendo (sd10). Así que fue bastante... fue... hubo un desequilibrio en mi salud mental (sd11) [...] pero luego acabé levant* ándome *y... cambié un poco la situación (sd12). [Mi esperanza viene más de mi valentía, por así decirlo. A veces tengo el valor de vivir más la vida. Aparte de estar bastante desanimada, como ocurre a veces, es... a veces tengo bastante valor para hacer algunas cosas (sd13).*(SD1).

Extracto 13:*No fue fácil, no fue fácil para nadie, todo el mundo sufrió, las consecuencias de la pandemia (sd3), así que somos supervivientes de un naufragio...(sd4) seguimos sobreviviendo, gracias a Dios...(sd5).*(SD14).

Extracto 14:*Así que espero ser feliz, perseguir todos mis objetivos (sd12). Levantarme temprano y salir a luchar,tener una vida normal, como cualquier otra persona (sd13).*(SD5).

Extracto 15:*[Esperanza significa] Intentar conseguir los puntos positivos de la vida... (sd9) «¡ah! tengo la esperanza de no tener problemas»...(sd10) pero hay que tener problemas en la vida para crecer...(sd11) saco [esperanza] de mí mismo y un poco de la fe que tengo en Dios... (sd12) es que mi vida ha sido un poco complicada desde pequeña, así que creo que he sido yo, así que necesitaba tener más fuerza para poder luchar (sd13).*(SD6).

## DISCUSIÓN

El estudio mostró que los sujetos produjeron diversos significados sobre la esperanza durante la pandemia, significados que fueron afectados por memorias discursivas, que no son conscientemente accesibles al sujeto, pero son constitutivas de discursos^(12,13)^. Los significados ya están en el mundo antes, en lo ya dicho, en lo pre-construido y vuelven en el decir^(15)^.

Los extractos 1 a 4 expresan significados de esperanza impregnados de memorias discursivas construidas por el discurso de la religiosidad/espiritualidad. La religiosidad puede definirse como las acciones, prácticas y creencias asociadas a una religión concreta. La espiritualidad tiene un significado más amplio, que implica la búsqueda personal de cuestiones esenciales para la vida y una conexión con lo sagrado o trascendental^(16)^. El Extracto 1 muestra la respuesta de SD5 a la pregunta - «¿Cómo describirías la esperanza en tu vida?». Las condiciones de producción del discurso son las de un usuario del servicio CAPS AD que ha vuelto recientemente al tratamiento y está sufriendo las consecuencias del abuso de SPA. En el sd1 se afirma que el significado de la esperanza es la ausencia de drogas. En sd8, la expresión «las cosas que solía hacer» silencia el significado «consumir drogas», ya que, desde la perspectiva de SD5, dicho consumo se considera inconveniente o inapropiado. Este silencio consiste en ocultar otros posibles significados que causan malestar, es decir, concebidos como inconvenientes^(13)^.

En un efecto metafórico de deslizamiento de significados, «mundo oscuro» significa «drogas» y la droga ciega a la persona y todo es imposible, mientras que el significado opuesto, «luz» sería la figura de Dios, es decir, cuando la persona sale de la oscuridad, «ve» y «constata» la existencia de Dios. En este sentido, la droga es la oscuridad y Dios es la luz. La paradoja entre oscuridad y luz remite al discurso religioso de que la figura de Dios es la solución al problema del consumo de SPA. Como afirma SD5, cuando ve a Dios, «todo es posible» (sd9) y «todo puede salir bien» (sd10).

En el extracto 2, el sentido de la esperanza se personifica en la figura de Jesús. En un cambio de significados, el significante «esperanza» adquiere un significado espiritual y religioso. El seguimiento discursivo - «hacer lo correcto»- silencia otros posibles significados de que lo correcto sería la creencia en Jesús y la voluntad, el deseo de tener fe. Este extracto expresa indicios de memorias discursivas vinculadas al discurso religioso y a la justicia/ética, en las que la esperanza pertenece al universo de la fe, de lo trascendente, pero también de la justicia/ética, es decir, de hacer lo correcto.

El extracto 3 muestra la respuesta de SD5 a la pregunta: «¿Cómo describirías la esperanza en tu vida?». El significado de esperanza adquiere el sentido de que Dios ayuda a las personas a no perder la esperanza. La idea de esperar una nueva vida, un nuevo comienzo (perspectiva de futuro) - surge en la paráfrasis «Nunca es demasiado tarde para empezar de nuevo» (sd14). Sd11 refuerza el discurso de la espiritualidad a través de la paráfrasis «Dios es amor en nuestras vidas». El pre-construido en otro lugar vuelve al discurso, demostrando que la espiritualidad expresa significados de amor, caridad, ayuda y esperanza.

SD14 afirma que la esperanza en su vida sería compartir este significado con otras personas. La sujeto enumera acciones espirituales que para ella están relacionadas con el significado de esperanza (rezar, interceder, hablar de Dios a las personas). Estas acciones forman parte de la dimensión conductual de la esperanza del Modelo de la Esperanza, que implica las acciones sociales, psicológicas, físicas o religiosas implicadas en el proceso de esperar y tener esperanza^(9)^.

El extracto 4 muestra la respuesta de SD5 a la pregunta: «¿Cómo describiría la esperanza en su vida?». En un efecto metafórico, el significado de esperanza se desplaza al significado de fe, en una transferencia contextual. Para SD6, fe y esperanza son «casi» sinónimos, es decir, para ella, el significado de esperanza está estrechamente relacionado con la creencia en la espiritualidad. Parafraseando el modelo de esperanza de Dufault y Martocchio^(9)^, existe un sentimiento de esperanza generalizada, es decir, no se refiere a un objeto específico de esperanza, sino más bien a un sentimiento generalizado de un «mañana mejor», un «futuro mejor» y la expectativa de cambios en el presente.

Las producciones discursivas religiosas/espirituales ejemplifican cómo se moviliza la dimensión afiliativa del proceso de esperanza^(9)^, que hace referencia a las relaciones con otras personas (vivas o muertas) o con un Ser Superior. Los extractos discursivos afirman que relacionarse con Dios es una fuente de esperanza para los sujetos del discurso. La espiritualidad forma parte de la dimensión comportamental de la esperanza^(9)^, considerando que los ritos y las prácticas religiosas, como la oración, la meditación, el culto y la participación en instituciones religiosas, por ejemplo, son acciones y comportamientos que pueden facilitar el surgimiento o el fortalecimiento de un estado de esperanza.

Los estudios^(17,18)^ corroboran la hipótesis de que la dimensión de la espiritualidad está estrechamente relacionada con la experiencia de la esperanza y muestran que las creencias espirituales son recursos internos significativos para obtener o mantener la esperanza. La espiritualidad se identificó como una fuente de esperanza en estos estudios^(17,18)^.

Los discursos anteriores evocan recuerdos discursivos del discurso religioso/espiritual. Los significados construidos sobre la esperanza están fuertemente atravesados por los significados de fe, creencia y espiritualidad. Como condición amplia de producción, cabe señalar que la población brasileña es mayoritariamente cristiana, de religiones católica o evangélica^(19)^. Las condiciones de producción se refieren a las circunstancias en que se producen los discursos y su relación para posibilitar el decir. Involucran el contexto socio-histórico, el imaginario y los aspectos ideológicos en los que se inserta el sujeto^(13)^.

La religión se presenta como una forma propia de lenguaje que tiene sus propios códigos y ritos y actúa como mediadora en el proceso de significación de la vida y de la realidad^(16)^. Las religiones, como prácticas sociales, forman parte de la constitución del sujeto y de la organización social, influyendo en la forma de ser y de construir significados de los sujetos^(16,20)^, por lo que se espera que la espiritualidad sea transversal a los discursos de los sujetos discursivos de este estudio.

Otro discurso destacado en este estudio fue el llamado a la solidaridad como forma de enfrentar las dificultades impuestas por la pandemia. La difusión transmitida por los discursos de las redes sociales y de los medios de comunicación fue que la pandemia proporcionaba una sensación positiva, es decir, la pandemia podía invocar la vivencia y los sentimientos de solidaridad en las personas, así como la percepción de que la adversidad pandémica desencadenaría sentimientos de valorar más la vida de las personas.

El discurso de la solidaridad se retoma mediante procesos de paráfrasis en los extractos discursivos 5 a 8. El extracto 5 forma parte de la respuesta dada por SD1 a la pregunta: «Cuando oye la palabra esperanza durante la pandemia, ¿qué le viene a la mente?». En un efecto metafórico, SD1 recuerda discursivamente que el color verde simboliza la esperanza. En el imaginario social, el color verde está relacionado con la naturaleza, la germinación y el renacimiento; la preservación del medio ambiente, el equilibrio y la armonía (en contraposición al ambiente de lucha y desacuerdo). Los segmentos discursivos «así, de solidaridad... más cariño, más cuidado... (sd4) la gente... debería ser más así (sd5). Hoy en día... hoy en día hay mucha pelea, mucho desacuerdo... (sd6)» revelan signos de un sentimiento de esperanza relacionado con un sentimiento de solidaridad. Existe una relación entre efectos de significado anclados en el Interdiscurso de valorar la solidaridad, es decir, ser solidario implica ser afectuoso y cariñoso, así como mantener un ambiente armonioso. El sujeto atribuye el significado de solidaridad al significante «esperanza».

El extracto 6 también enuncia significados anclados en el Interdiscurso de la solidaridad, en el que la pandemia hace que las personas sean más solidarias y valoren más las cosas que pierden. Para SD4, la esperanza en el periodo pandémico sería que hubiera más «humildad» entre la gente. Se puede ver un fallo cuando SD4 elige el significante «humildad» y no otros posibles, como «humanidad o solidaridad». Sin embargo, en el extracto discursivo, el significante «humildad» adquiere otro significado cuando se inscribe en esta formación discursiva particular - el significado de solidaridad, que es diferente de su significado literal que, según el diccionario Aurélio, significa «modestia, sencillez». Para AD, este efecto de significado demuestra cómo las palabras no tienen un significado literal y original; el significado puede ser desplazado y dotado de otro efecto, es decir, puede circular entre diferentes regiones discursivas^(21)^.

La solidaridad surge como respuesta de la sociedad a la pandemia, recurso que puede ser movilizado individual y/o colectivamente por empresas, grupos organizados por la sociedad civil, movimientos sociales y poderes públicos^(22)^. En este estudio, el significante «solidaridad» se relaciona con el significado de «solidaridad filantrópica», expresado por el comportamiento de individuos o pequeños grupos de realizar acciones voluntarias, asistenciales y caritativas, así como de mantener una postura empática.

El significante «esperanza», cuando pertenece al universo semántico del significante «solidaridad», se refiere a la dimensión afiliativa del Modelo Esperanza^(9)^. La ayuda mutua, la reciprocidad y la preocupación por los demás forman parte del proceso de la esperanza, ya que nos relacionamos con los demás y podemos reforzar la esperanza a través de las relaciones.

El extracto 7 expresa la sensación de que la pandemia desencadenaría algo bueno para la humanidad, es decir, momentos de reflexión para que la gente se detenga y se tome la vida «con más calma». Según SD12, se produjo una romantización durante la pandemia, es decir, experimentar momentos difíciles puede animar a la gente a valorar más la vida. En un efecto deslizante de significados, en sd4 «la gente debería parar y frenar un poco» tiene el efecto de ralentizar, tranquilizar, hacer una pausa, lo que, en la situación dada, implicaría valorar la vida (sd5). Sin embargo, en sd6, SD12 concluye que este cambio esperado no se ha producido; al «volver todo a la normalidad», «la humanidad vuelve a la normalidad», es decir, vuelve a un ritmo rápido, un ritmo que no conduce a la valorización de la vida. Otra pista importante en este apartado discursivo es la «visión romantizada» mencionada en sd7, que se refiere a los recuerdos discursivos difundidos durante la pandemia, de un buen periodo para la humanidad, es decir, de valorar la vida frente a la adversidad pandémica.

Durante el período pandémico, las redes sociales online y los medios de comunicación expusieron el imaginario social diverso, es decir, una parte de la población favoreció la divulgación de informaciones sobre la pandemia, otra enfatizó el potencial peligroso del virus y las pérdidas causadas por las complicaciones del COVID-19, y otra expresó experiencias positivas derivadas de la pandemia, como relatos de actos solidarios y caritativos, experiencias individuales de superación y experiencias personales positivas de reinvención de hábitos y rutinas, según la literatura^(23)^.

Los extractos 8 a 11 enuncian efectos de sentido afectados por memorias discursivas derivadas del modelo de enfermedad, en el que los síntomas y la medicación ocupan un lugar central en el proceso terapéutico, basado en la premisa de «enfermedad-cura» y «problema-solución». En el contexto del consumo de alcohol y otras drogas, la dependencia química es una enfermedad y sólo puede «curarse» mediante la abstinencia^(24)^. En el contexto de los trastornos mentales y los derivados del consumo de SPA, el trastorno mental es una enfermedad que puede tratarse con medicación, constituyendo un problema que tiene una causa y requiere una solución, en este caso, la medicación.

El paradigma racionalista de «problema-solución» fue hegemónico hasta las primeras experiencias de Reforma Psiquiátrica en los años 60 y las prácticas subvencionadas en Salud Mental y Psiquiatría. En este paradigma, el problema dado era la enfermedad y la solución esperada era la cura. La terapia en esta lógica es considerada como un proceso que vincula el diagnóstico con el pronóstico esperado - una cura. Con las primeras iniciativas de Reforma y el avance del conocimiento en el área de la salud mental, se comprende que las personas con trastornos mentales tienen una experiencia integral, compleja y concreta que no puede reducirse a la lógica «problema-solución»^(25)^. Para trabajar en la práctica con los usuarios de los servicios especializados de salud mental, es necesario superar el paradigma problema-solución y pasar a considerar que, en el contexto de los trastornos mentales, el sufrimiento psíquico es una experiencia subjetiva y compleja en un contexto social y, en el contexto del consumo de alcohol y drogas, existen enfoques que reconocen la autonomía, el protagonismo y la singularidad de los usuarios, como el enfoque de Reducción de Daños^(25,26)^. De esta forma, los usuarios son considerados sujetos sociales y actores del cambio y de la reconstitución del sentido de la vida^(25)^.

En el extracto 9, SD5 espera que no haya más «recaídas», y este significante revela señales de un discurso basado en el modelo de enfermedad y en la prevención de recaídas. Desde esta perspectiva, la dependencia del SPA es una enfermedad y, por lo tanto, el objetivo es lograr una cura, es decir, la abstinencia. La recaída significa volver a consumir, lo que debe prevenirse en este modelo^(24)^.

Los extractos anteriores expresan la dimensión cognitiva del proceso de esperar y tener esperanza, cuando los sujetos discursivos elaboran, interpretan, juzgan y enumeran sus objetos de esperanza, definiendo prioridades, por ejemplo^(9)^. SD2 atribuye el significado de esperanza al deseo de «recuperación» y SD7 destaca la curación como su principal y primer objeto de esperanza. SD5 define la abstinencia de SAF como su objeto de esperanza, manteniéndose libre de recaídas.

En el extracto 10, SD1 señala la contradicción y crítica de la posición social del sujeto discursivo en un determinado momento de su vida, es decir, ser traficante de drogas y consumidor de SPA al mismo tiempo. El significante «hervía» en SD2 denota significados de ebullición, efervescencia, burbujeo, que enuncia significados de consumo intenso de drogas, especialmente durante la pandemia. Destaca en sd3 - «Tengo que vivir de la medicina» - el enunciado de significados retoma las memorias discursivas del modelo de enfermedad, en el que los medicamentos son la principal solución terapéutica al sufrimiento psíquico^(24)^. El discurso enfatiza no sólo la dependencia del PAS, sino también de los fármacos, que sostienen la gestión de la vida del enunciador.

En el extracto 11, SD13 se refiere a no tener esperanza debido a la depresión «instalada», teniendo este significante un efecto metafórico de transferencia contextual, es decir, significados de «materializado», «instalado», «diagnosticado». Para SD13, el hecho de que su trastorno mental esté «instalado» condiciona y determina su experiencia de la esperanza. Las marcas y trazos lingüísticos derivan efectos metafóricos del hecho de que la usuaria tendría que «hacer todo lo posible para salir» de la depresión, para curarse, para que en el futuro, «en otro momento», pudiera tener sueños y metas.

La condición del diagnóstico de depresión, o la ausencia del mismo, es un factor determinante en la capacidad de tener esperanza. La dimensión contextual influye en el proceso de esperanza, ya que las circunstancias y situaciones de la vida interfieren en la capacidad del sujeto para mantener la esperanza^(9)^. La condición de producción estricta de empeoramiento de la depresión y la experiencia de duelo de SD13 debido a la pandemia desencadenaron sentimientos de desesperanza.

La suposición de que la desesperanza y la esperanza no son antagónicas refuerza la percepción de que una misma situación puede dar lugar a oportunidades para la esperanza o la desesperanza, dependiendo de cómo el sujeto perciba, interprete y reaccione ante el contexto. Los contextos que denotan desesperanza pueden despertar nuevos objetivos, revisar planes y estrategias^(9)^.

Los extractos 12 a 15 enuncian significados de esperanza que se ven afectados por memorias discursivas preconstruidas del discurso de la resiliencia. SD1 habla de «desequilibrio de la salud mental» al definir el significado personal de la esperanza en tiempos de pandemia. El significante «desequilibrio» denota la derivación de los efectos metafóricos de los significados de que el sufrimiento psíquico le hace perder el equilibrio y caer, a pesar de que el sujeto discursivo reconoce su «valentía» como fuente de esperanza que le motiva a «levantarse» y «cambiar la situación». Estos significados apuntan a la valorización de características internas y recursos personales de afrontamiento y resiliencia, buscando superar las dificultades del período pandémico.

Recordando las estrictas condiciones de producción de SD1 en que fue enunciado el discurso, se destaca el lenguaje no verbal de la entrevistada, expresado por su postura cansada y desanimada. Existe una contradicción y ambivalencia entre el reconocimiento del «coraje» de SD1 y el sujeto-posición del desánimo. La esperanza está estrechamente vinculada a la condición de incertidumbre y se caracteriza por ser una experiencia humana que puede oscilar, alternando entre momentos de alta y baja esperanza. Algunas respuestas afectivas a la incertidumbre son los sentimientos de ansiedad, vulnerabilidad, preocupación y tristeza^(9)^.

En el extracto discursivo 13, SD14 utiliza la metáfora figurativa para comparar a la sociedad con un barco que se hunde durante la pandemia, denotando significados de destrucción y aniquilación, pero con supervivientes. El sujeto discursivo enfatiza la empatía al afirmar el sufrimiento compartido y experimentado colectivamente por las personas durante este periodo. La elección del significante «supervivientes» sugiere a su vez significados de resiliencia. En las estrictas condiciones de producción en las que se emitió el discurso, SD14 cree que es posible adaptarse y superar las dificultades impuestas por la pandemia, en otras palabras, «sobrevivir al naufragio».

En el extracto 14, el sujeto del discurso atribuye significados de resiliencia y esfuerzo al significante «esperanza», elucidando el funcionamiento de la dimensión conductual de la esperanza, que implica las acciones movilizadas por los sujetos para lograr algo significativo para sí mismos y para los demás^(9)^. El sujeto discursivo desarrolla estrategias relacionadas con el campo de acción (levantarse temprano, ir a por todas, luchar), que le ayudarán a alcanzar el resultado esperado. Sd13 enuncia la marca lingüística de la paráfrasis, cuando SD5, en su dicho - «Levantarse temprano e ir a la lucha»- parafrasea el dicho popular - «Al que madruga Dios lo ayuda»-. El mecanismo de paráfrasis adopta significados preconstruidos de valoración de la dedicación y el esfuerzo.

Para SD5, estos significados se atribuyen a «tener una vida normal», eligiendo el significante «normal» y no otros. Este fenómeno en AD se denomina olvido n.2, en el que el sujeto del discurso favorece y selecciona ciertas palabras en lugar de otras^(12)^. La elección de decir una determinada palabra y no otra tiene un significado que se relaciona con las condiciones amplias y estrictas de producción, las históricas y las ideológicas^(13)^. Para SD5, «normal» está relacionado con el trabajo duro, la funcionalidad y la lucha.

Se puede observar que el significado de resiliencia es transversal a los extractos anteriores, que se entiende como un fenómeno complejo que ha sido estudiado en los últimos años por diversas áreas, como las Ciencias Humanas y las Ciencias de la Salud, y conceptualizado por diversos autores^(27,28)^. La resiliencia puede definirse como el proceso de hacer frente a los desafíos o adversidades y como la capacidad de desarrollar respuestas adaptativas para obtener los resultados esperados. Es un proceso que se desarrolla a lo largo de la vida y en el que intervienen recursos de afrontamiento personales, interpersonales y sociales^(29)^. El significado de resiliencia, pre-construido previamente y en otros lugares, es retomado por los sujetos y materializado en sus discursos cuando describen su experiencia de esperanza durante la pandemia de COVID-19.

En el extracto 15, el significado revelado es el de la valoración de la superación de la adversidad, durante la respuesta a la pregunta: «¿Qué significa para ti la esperanza?». El significado revelado es el de resiliencia y superación, ya que el sujeto moviliza recursos internos (fuerza/resiliencia personal) y externos (un poco de fe) para empoderarse ante la adversidad. Sd11 - «hay que tener problemas en la vida para crecer»- presenta la clave lingüística de la paráfrasis, entendida como el retorno de la memoria en el enunciado^(13)^. Esta secuencia discursiva retoma significados pre-construidos en otros lugares, como que «el sufrimiento hace crecer» y que las dificultades permiten el aprendizaje y los cambios positivos.

Para AD, al analizar la constitución de un discurso, analizamos los discursos que hay en él, es decir, el Interdiscurso^(30)^. El sd11 retoma el discurso socialmente difundido, por lo tanto, de formaciones ideológicas de valorización de la adversidad. Para el sujeto, la esperanza adquiere el significado de superar las dificultades de la vida y el sd13 - «necesitaba tener más fuerza para poder luchar» - refuerza la valorización del esfuerzo, de la capacidad y de la competencia individuales para enfrentar la adversidad.

Para AD, las condiciones de producción se refieren a una suma de variables que conforman la situación en la que se emite el discurso, considerando el contexto socio-histórico, el contexto personal y el lugar desde el que habla el sujeto^(30)^. En este extracto, se observa que frente a un macrocontexto (pandemia) y un microcontexto (historia de vida personal), el sujeto ha experimentado situaciones vitales difíciles - «mi vida ha sido un poco complicada desde niño»- que le han empujado a «luchar» contra la adversidad.

Se entiende que la capacidad de aprender de afrontar el problema en el pasado o en el presente favorece una resolución futura del problema, pero no la exclusión del problema en el pasado, presente y futuro. Por lo tanto, el objetivo es esperar competencia y capacidad para resolver problemas futuros. Así, este contexto implica la dimensión temporal de la esperanza, en la que la experiencia del pasado y del presente subvenciona el futuro, para posibilitar la competencia y la habilidad^(9)^.

En cuanto a las limitaciones de este estudio, pueden estar relacionadas con el período de recolección de datos durante la pandemia de COVID-19, considerando las estrictas condiciones de producción en que se inició la vacunación y el fin de esta enfermedad como emergencia de salud pública. Otra limitación corresponde al predominio de participantes del sexo femenino en el estudio en relación a la percepción del sentimiento de esperanza, exigiendo una ampliación del estudio con la población masculina.

Los resultados de este estudio contribuyen a una escucha más empática y sensible de la vivencia de la esperanza a partir de los discursos de los usuarios de los servicios especializados de salud mental durante el período de pandemia de COVID-19, lo que puede favorecer tanto a los profesionales de la salud, especialmente enfermeros, como a los usuarios de los servicios de salud mental, a (re)conocer la esperanza propia y ajena como fuentes promotoras de salud mental.

## CONCLUSIÓN

Los discursos producidos sobre la esperanza evocan memorias discursivas construidas antes y fuera del sujeto. Los significados de la esperanza fueron permeados por los siguientes discursos: religioso/espiritualidad, solidaridad, modelo de enfermedad y resiliencia. Creemos que es importante formar a los profesionales de la salud, especialmente a las enfermeras, que trabajan en CAPS y CAPS AD, para que capten los significados construidos de la esperanza y los reconozcan como un recurso terapéutico que forma parte de los cuidados. Se destaca la importancia de reconocer la promoción de la esperanza como intervención en la práctica clínica y asistencial.
